# mHealth Series: Factors influencing sample size calculations for mHealth–based studies – A mixed methods study in rural China

**DOI:** 10.7189/jogh.03.020404

**Published:** 2013-12

**Authors:** Michelle Helena van Velthoven, Ye Li, Wei Wang, Xiaozhen Du, Li Chen, Qiong Wu, Azeem Majeed, Yanfeng Zhang, Josip Car

**Affiliations:** 1 Global eHealth Unit, Department of Primary Care and Public Health, Imperial College London, London, UK; 2 Department of Integrated Early Childhood Development, Capital Institute of Paediatrics, Beijing, China

## Abstract

**Background:**

An important issue for mHealth evaluation is the lack of information for sample size calculations.

**Objective:**

To explore factors that influence sample size calculations for mHealth–based studies and to suggest strategies for increasing the participation rate.

**Methods:**

We explored factors influencing recruitment and follow–up of participants (caregivers of children) in an mHealth text messaging data collection cross–over study. With help of village doctors, we recruited 1026 (25%) caregivers of children under five out of the 4170 registered. To explore factors influencing recruitment and provide recommendations for improving recruitment, we conducted semi–structured interviews with village doctors. Of the 1014 included participants, 662 (65%) responded to the first question about willingness to participate, 538 (53%) responded to the first survey question and 356 (35%) completed the text message survey. To explore factors influencing follow–up and provide recommendations for improving follow–up, we conducted interviews with participants. We added views from the researchers who were involved in the study to contextualize the findings.

**Results:**

We found several factors influencing recruitment related to the following themes: experiences with recruitment, village doctors’ work, village doctors’ motivations, caregivers’ characteristics, caregivers’ motivations. Village doctors gave several recommendations for ways to recruit more caregivers and we added our views to these. We found the following factors influencing follow–up: mobile phone usage, ability to use mobile phone, problems with mobile phone, checking mobile phone, available time, paying back text message costs, study incentives, subjective norm, culture, trust, perceived usefulness of process, perceived usefulness of outcome, perceived ease of use, attitude, behavioural intention to use, and actual use. From our perspective, factors influencing follow–up were: different caregivers participating in face–to–face and text message survey, sending text messages manually, participants responding incorrectly, and technical issues. Participants provided several recommendations for improving follow–up and we added our views to these.

**Conclusions:**

This is the first study to evaluate factors influencing recruitment and follow–up of participants in an mHealth study in a middle–income setting. More work is needed to assess effectiveness of our suggested strategies. This work would improve evaluation of mHealth interventions.

mHealth, or mobile health, has the potential to improve the delivery of health care and improve health worldwide. However, there is limited thorough mHealth evaluation and thus insufficient evidence to implement and scale–up effective mHealth interventions [1,2]. An important issue for mHealth evaluation is the lack of information for sample size calculations. Sample size calculations are influenced by both recruitment and follow–up of participants. When the sample size targets are not met, this can lead to an underpowered study in which differences between groups are statistically non–significant. Extending the recruitment period increases costs and introduces logistical issues. In addition, when the number of participants who are recruited is low and loss to follow–up is high, the risk of selection bias and retention bias is considerable, which limits generalizability of results [3].

Problems with recruitment and retention are common; a review of 73 randomised controlled trials reported that only 40 (55%) achieved their original recruitment target [4]. A systematic review of 45 randomised controlled trials that used activities to improve recruitment found the following strategies successful: telephone reminders to non–responders, use of opt–out rather than opt–in procedures for contacting potential participants, and open designs where participants know which intervention they receive [5]. A systematic review including 28 population–based cohort studies of interventions to improve retention of participants found that incentives, reminder letters or call, and alternative data collection modes demonstrated a benefit [6]. However, while reporting of recruitment and follow–up of participants in studies has been improved by checklists, such as CONSORT for randomised trials, reporting is still often not described with sufficient level of detail [7].

mHealth–based studies face specific difficulties with reaching their target sample size. mHealth interventions are complex in their nature as technology interacts with health system designs and people. Selection bias occurs when a large number of people do not use mobile phones and when consent–rates are low. Despite the ubiquity of mobile phones in low– and middle–income countries, there may be differences in use by gender, age, education and income [8,9]. Individual mHealth data collection has shown to face several challenges [10]. Effective follow–up is particularly important for studies that use text messaging [1] as text message data collection studies have reported variable response rates [10–21]. We had little information available to calculate an accurate sample size when conducting our mHealth data collection studies on child health in Zhao County, rural China.

Mobile phones are commonly used in both urban and rural China and the Chinese government has introduced many text messaging public health education programs, including programs for child health [22]. However, there have only been a few mHealth studies in China and the applicability of mHealth to the Chinese health care system has not been assessed [22]. China has a three–tier health care system with usually a general hospital and a maternal and child health hospital at county level, one hospital in each township and one clinic in each village [23]. Health workers in township hospitals are the main provider of antenatal and postnatal care, and vaccination [24]. Almost all women deliver in the township or county hospital and health workers record names of caregivers and their children after birth [25]. Township and county level health workers train and supervise village doctors [26,27]. Village doctors provide general primary health care at village level, including some maternal and child health care. Education and training of village doctors varies, but usually they have at least primary school or junior high school and short basic medical training. Village doctors live in the communities they serve and have a good relationship with villagers.

This study was part of a larger mHealth project and in the first paper of this mHealth series, we described the aims of the project, field site in China, and methodology [28]. In the current paper, we aimed to explore factors that influence sample size calculations for mHealth–based studies and to suggest strategies for increasing the participation rate. We used our experiences with recruitment and follow–up of participants in an mHealth text messaging data collection cross–over study. Generic lessons can be learned from our experiences and they will help future mHealth studies with estimating their sample sizes.

## METHODS

### Overview of methods

Recruiting and following up participants in an mHealth data collection cross–over study was challenging (methodology described in the first article in this mHealth series) [28]. We used a mixed methods design with the purpose of explaining our findings from the cross–over study [29]. We aimed to explore factors influencing recruitment and follow–up and to suggest strategies to improve participation.

In this methods section, we started with a brief description of the cross–over study. Then we described the process of recruitment, number of caregivers recruited, and methods for evaluating recruitment. Finally, we described the process of follow–up, number of participants followed up and methods for evaluating follow–up.

### Cross–over study

In the cross–over study, we randomised caregivers of children under five at village level into group 1 and group 2. The aim of the cross–over study was to determine the validity of an mHealth text messaging survey. We compared the traditional face–to–face survey method to the new text messaging survey method. The study took place in Zhaozhou Township, Zhao County, Hebei Province, China in March 2013. The detailed characteristics of the sample of participants and results on the outcomes of the cross–over study will be reported elsewhere (unpublished).

### Recruitment

**Process of recruitment.** The Zhaozhou Township hospital and four affiliated vaccination clinics provided a list of names (referred to as the “name list”) with names of children and their caregivers, children’s date of birth and sometimes phone numbers of caregivers in Zhaozhou Township. We asked township hospital and county hospital doctors to contact village doctors and to arrange a time for recruitment. Village doctors were asked to gather in their village clinic all caregivers of young children who lived in their village. Initially, we asked village doctors to use their own list with names of caregivers for gathering caregivers (we thought that they had their own name list), but they did not have their own name lists. Therefore, we gave village doctors the name list, and asked them to validate the names.

Before we arrived in the village, we asked village doctors to make an announcement with loudspeakers when possible. In addition, we asked village doctors to make phone calls to caregivers and to go to caregivers’ houses to invite them if no phone numbers were available. We visited villages during the day and late afternoon to recruit parents who were working during the day (we were unable to go to villages in the evening). We also asked caregivers to notify their neighbours, we asked people on the street and we went to places that caregivers visited.

We provided village doctors a small compensation (¥ 50, about £ 5.3, € 6.2, US$ 8.2, for recruiting 55 caregivers) for their efforts. When village doctors recruited more caregivers, the amount they received increased with ¥ 10 (¥ 60 for 55–65 caregivers, ¥ 70 for 66–75 and so on). As recruitment was challenging, we decided to visit villages in group 1 for the second time to recruit caregivers (this was not possible in group 2). For the second visit, we increased the incentive to ¥ 10 for every four caregivers village doctors recruited.

We included caregivers who had a child younger than five, used a mobile phone and were able to text message. We gave a towel (worth ¥ 5, about £ 0.52, € 0.62, US$ 0.82) to caregivers for participating in the face–to–face interview. In addition, we found during the fieldwork that caregivers were interested in child health information and we provided a health information calendar that we developed in a previous study in 2012 [30].

**Number of caregivers recruited.** The name list had 4170 children under five at the time of the study. We randomised 16 villages with 1600 children under five into group 1 and 30 villages with 2570 children under five into group 2 (our study design required more children to be allocated to group 2). To estimate the number of caregivers of children we would be able to recruit, the only estimate we had available was recruiting 70% of caregivers for previously conducted household surveys. Taking this 70% into account, we would be able to recruit and include 2920 caregivers in 46 villages (1120 participants in group 1 and 1800 in group 2). However, we only recruited 1026 caregivers in 42 villages and we had to exclude 12 caregivers for the following reasons: the child of one caregiver just reached the age of five, we did not send text messages to three caregivers in group 1 because of an administrative mistake, and we could not identify which child belonged to the text message responses for eight caregivers (which we only realized after the study). Those eight caregivers were four caregivers who gave the same mobile phone number as four other caregivers (they belonged to only four different families and gave one phone number per family). Therefore, of the 4170 names of children on the name list, we included 1014 (24%); 371 in group 1 and 643 in group 2.

**Evaluation of recruitment.** We conducted semi–structured interviews with village doctors who recruited participants and we added our views. The detailed methodology of the interviews was described in the first paper in this mHealth series [28].

***Data collection.*** Two trained female researchers (WW and XD) conducted the interviews in Chinese at the end of the second visit to villages (the aim of this visit was recruitment in villages in group 1 and follow–up in villages in group 2). The interviews were carried out in a quiet private room in the village clinic. When the village doctor gave permission, the interview was recorded, and notes were taken to record non–verbal communication. We used probing questions (open–ended questions; starting with how, why, what etc.) to follow up on the questions in the interview guide [31].

***Sample.*** We included ten village doctors who recruited participants, seven males and three females. The village doctors were from six different villages in group 1 (Table S3 in Online Supplementary Document(Online Supplementary Document)) and from four different villages in group 2 (Table S4 in Online Supplementary Document(Online Supplementary Document)). Their age ranged between 29 and 63 years and all completed a secondary school.

***Data analysis.*** We used thematic analysis [32] and aimed to provide a description of the entire data set that reflected the important themes in the interviews. Two Chinese researchers (YL and XD) analysed the data in Chinese and independently translated the main findings into English, compared them and through discussion developed the English translation of the main findings. The findings were discussed and further analysed with help of a researcher fluent in English (MV). A bilingual translator translated the main findings from English back into Chinese, we compared this with the original Chinese and revised the English where needed [33]. In addition, we added our own views and experiences to the themes that we found in the interviews. We clearly identified which views were from village doctors and which views were ours.

### Follow–up

**Process of follow–up.** In group 1, our interviewers obtained informed consent from caregivers and administered the face–to–face survey in the village clinic. A day after the face–to–face interview, we sent participants the text messages survey questions (which participants could answer at a place of their convenience). In group 2, interviewers obtained informed consent in the village clinic and then we sent participants the text messages. We asked caregivers who responded to at least the first survey question in text message 4 (question about whether the child had diarrhoea in the past two weeks) to visit the village clinic again for the face–to–face survey the day after the text message survey ended.

The face–to–face and text messaging survey both had 17 overlapping questions on care–seeking for childhood diarrhoea and pneumonia signs and symptoms that we compared between the methods. The face–to–face survey had additional questions on demographics, the household and mobile phone use. The text messaging survey had three follow–up questions and two additional questions: one on agreement to participate and one on the relationship between the participant and the child. In the face–to–face survey, trained interviewers recorded participants’ answers with smartphones [34]. In the text message survey, we manually sent text messages to participants using a Chinese text message system [28]. The number of the text message system contained 16 digits (1065–5059–1091–1763). This was a special number, because normal Chinese mobile phone numbers have only 11 digits.

For the face–to–face survey, as reported in the recruitment section, we gave a towel and health information calendar to participants. For the text message survey, we paid back the text message costs to all participants who responded and provided ¥ 5 if participants completed the text message survey. We gave participants two days to respond to the text message questions. We sent two reminder text messages (nine and 24 hours after the first text message). We sent text messages and made phone calls to participants in group 2 who had to return to the village clinic for the face–to–face interview.

**Number of participants followed up.** Of the 1014 participants in the cross–over study, 662 (65%) responded to the first text message question about willingness to participate in text message 2. A total of 538 (53%) responded to the first survey question in text message 4 (question used for data equivalence sample size calculation), which was less than the 56% response rate we assumed. A total of 356 (35%) participants completed the text message survey (we could not estimate how many participants would complete the survey).

In group 1, of the 371 participants who were interviewed face–to–face, 233 (63%) responded to text message 2, 189 participants (51%) responded to text message 4 and 137 participants (37%) completed the text message survey. In group 2, of the 643 participants who provided informed consent during the first visit, 429 (67%) responded to text message 2, 349 participants (54%) responded to text message 4, and 219 (34%) completed the survey. We invited the 349 participants who responded to text message 4 to come to the village clinic for the face–to–face interview and assumed 70% to come, but 302 (87%) came. Even an additional five participants came who had not responded to text message 4 (we had not asked them to come).

**Evaluation of follow–up.** We interviewed participants who did not respond to the text messaging survey (referred to as “non–responders”), participants who responded to text message 2 but did not complete the text message survey (referred to as “non–completers”), and participants who completed the text message survey (referred to as “completers”). In addition, we described our experiences with follow–up. We asked participants for their recommendations to improve follow–up and added our views to these. We provided a detailed description of this methodology in the first paper in this mHealth series [28].

***Data collection.*** We interviewed participants via telephone interviews and face–to–face. The two supervisors of the cross–over study (WW and XD) interviewed non–completers in group 2 when they returned to the village clinic for the face–to–face interview. In the week after completing the fieldwork, we conducted telephone interviews with completers and non–responders in both groups, and non–completers in group 1, because we could not interview these participants face–to–face. Four team members (WW, XD, YL, and QW) conducted the telephone interviews. They called participants at a time convenient for participants. When the phone call was unanswered, they called participants back up to three times. The interviewers used a pen–and–paper questionnaire to record the interview. The interviews were structured and we combined closed–ended and open–ended questions (with follow–up probing questions). We asked non–responders for their views on the text messaging method and non–completers and completers for their views on the face–to–face and text messaging methods.

***Samples.*** We used simple random sampling with SAS version 9.2 (SAS Institute Inc, Marlow, UK) to select random samples of non–responders, non–completers, and completers. We randomly selected 125 non–responders out of 352 non–responders. We could not reach 57 non–responders, we reached 68 non–responders and we included 62 non–responders: 55 mothers (89%) and 7 fathers (11%). We had to exclude six non–responders: four did not want to participate and two quit before giving an answer to the first question. We randomly selected 93 non–completers out of 306 non–completers. We could not reach 35 non–completers and we included 58 non–completers who answered questions: 42 mothers (72%), 12 fathers (22%), 2 grandmothers (3%) and 2 grandfathers (3%). Of those 58 included non–completers, 56 finished the interview and two non–completers quit the interview before the end, but gave answers to questions. We randomly selected 110 completers out of 356 completers. We could not reach 37 completers and we included 73 completers: 58 mothers (80%), 13 fathers (18%), 1 grandmother (1%) and 1 grandfather (1%). Of those 73 completers, 68 finished the interview and six caregivers quit the interview before the end, but answered questions.

***Data analysis.*** We calculated proportions for the closed–ended questions. We conducted a thematic analysis for the open–ended questions. Two Chinese researchers (YL and WW) independently read through the data several times, identified the main themes in the data and summarized the main results in Chinese. The approach for translation of the results was similar to the approach for translation of the results of semi–structured interviews with village doctors.

During analysis of the qualitative data, we found that our themes had overlap with variables in the Technology Acceptance Model [35] and modified versions of this model [36–39]. Therefore, we organized our data according to variables in these models. The Technology Acceptance Model proposes that a person’s acceptance of a technology is determined by its perceived usefulness and perceived ease of use [35]. The model predicts that ease of use and usefulness will influence a person’s attitude toward, intention to use, and acceptance of the technology. Consequent factors of perceived usefulness and ease of use are attitude, behavioural intention to use, and usage. In our context, perceived usefulness was participants’ perception that the survey methods enhanced the process of participation in the study and that participation had a useful outcome. Perceived ease of use was a participant’s perception that the survey methods were free of effort. Additional variables proposed in modified models are variables that influence perceived ease of use or usefulness. These variables depend on the context, and include prior usage, gender [40], trust, perceived financial costs [41], culture [42] and subjective norm [39]. Subjective norm is an individual’s perception of the degree to which important other people approve or disapprove of behaviour.

## RESULTS

### Overview of results

First, we described factors influencing recruitment and recommendations for ways to recruit more caregivers based on interviews with village doctors and on our own experiences. Second, we described factors influencing follow–up and recommendations for improving follow–up based on interviews with non–responders, non–completers, and completers, and on our own experiences.

### Recruitment

We provided a summary of village doctors’ and our views on factors influencing recruitment and ways to recruit more caregivers; a detailed description can be found in Online Supplementary Document(Online Supplementary Document).

**Factors influencing recruitment.** We found several factors influencing recruitment related to the following themes: (i) experiences with recruitment, (ii) village doctors’ work, (iii) village doctors’ motivations, (iv) caregivers’ characteristics, and (v) caregivers’ motivations.

***Experiences with recruitment.*** Generally, we did not have problems with reaching villages, but road problems did not allow us to visit one village for the second recruitment round in group 1. Although we carefully organised our fieldwork, it was difficult to reschedule when there was a problem. Most village doctors were available to help us with gathering caregivers, but when they were not helpful this often resulted in only finding a small number of caregivers. Some village doctors said that they did everything they could to recruit caregivers, while others said that because participation in the study was voluntary, they helped but did not do their best. Village doctors were able to recruit more caregivers when the name list and phone numbers of caregivers were available. Village doctors did not have their own name list, so we relied on the name list from the township hospital. The township name list was not accurate, because children on the list did not always seem to live in villages and we found children who were not on the name list of villages. The township hospital had phone numbers of caregivers for a number of villages and in addition some village doctors had phone numbers of caregivers they knew. However, it was common that there were mistakes in the phone numbers due to wrong recording, or phone numbers were no longer in use. Many parents were not home during the time that we visited the villages (during the day), while grandparents were often home and took care of children. Village doctors said that our study selection criteria made it harder for them to recruit caregivers. Most village doctors used the villages’ loud speaker to gather caregivers and found this convenient. Some village doctors also made phone calls to caregivers and we called caregivers when village doctors were not able to do this. Only few village doctors visited caregivers’ houses, because this was time–consuming and they did not always know where caregivers lived. Other recruitment methods included going on the street and to places where caregivers often came, and asking caregivers to notify others. The effectiveness of the different strategies depended on the specific context of the village.

***Village doctors’ work.*** Village doctors’ work included treating patients and selling medicines. We found that village doctors also did other work to increase their income. Some village doctors had their own village clinic, while others shared their clinic with other village doctors. At the time of our study (March), village doctors were not busy and mainly worked in the morning and evening. Village doctors had previous experiences with recruiting caregivers for vaccination or had participated in our previous studies.

***Village doctor’s motivations.*** Village doctors did not always understand the aim of the research well and sometimes found it not useful. Village doctors did not experience delays in their normal work, but during busy times in the year, they would not participate when the study interfered with their work. Village doctors often said not to mind about the compensation we provided for their time, but in our experience some did mind. Other motivations were that village doctors wanted to work for villagers, follow orders from hospital doctors and cooperate with our research team.

***Caregivers’ characteristics.*** Caregivers were often busy with work and did not have time to participate when they had to earn their income. Village doctors thought caregivers’ education was not always sufficient to understand the survey questions, but in our experience most caregivers were able to understand our questions. Parents could usually text message, but grandparents could often not.

***Caregivers’ motivations.*** Township hospital doctors’ explanation of the research was not sufficient to inform village doctors well. Many village doctors did not have a good understanding of the study. Thus, village doctors did not seem to explain the study well to caregivers when asking them to come to the village clinic. Caregivers did not always understand why they had to come, and found it not useful when their child was not ill. When we explained the study to caregivers when they were in the village clinic, still village doctors thought that many caregivers did not understand the aim well. However, we found that most caregivers understood what we were doing. Village doctors felt that caregivers found it difficult to trust us, because caregivers were concerned about being misinformed or deluded. Sensitive questions about income and expenses were perceived useless and caregivers did not understand why these had to be asked, because the study was about child health. Village doctors thought that many caregivers came for the reward (towel). We felt that caregivers were interested in good health information.

**Recommendations for ways to recruit more caregivers.** More caregivers could be recruited when the name list and phone numbers were given in advance. Village doctors said that if we would include all caregivers, then they would be able to recruit more caregivers, but this would not be feasible for text message studies. Village doctors thought that it would be better recruit caregivers earlier on the day, because then more caregivers had time. In addition, we think going to the villages in the evening may be a good strategy too. However, village doctors’ and interviewers’ working hours would have to be taken in consideration. Continuing to use the village’s loudspeakers, make phone calls and send text messages to caregivers were recommended. Village doctors were willing to visit caregivers’ houses when they had more time available, but this would be a time–consuming approach. Village doctors recommended giving caregivers more money than caregivers’ could earn, but this would not be desirable and too costly. Another recommendation was to give a free health test for children, but we consider this only to be appropriate when this is required for a study. It was mentioned to bring a doctor who could give health information. We think that this may increase trust of village doctors and caregivers, but to provide health information, a more cost–effective solution may be to send health information text messages. Moreover, to address factors that negatively influenced recruitment, we suggest to develop and test new information materials for village doctors and caregivers, omit sensitive questions from survey and tailor recruitment strategies to the specific context of villages.

### Follow–up

First, we described factors influencing follow–up. Second, we presented recommendations for improving follow–up.

**Factors influencing follow–up.** We described participants’ views on factors influencing follow–up reported by non–responders, non–completers, and completers, followed by researchers’ views.

***Non–responders.*** **Table 1** shows the quantitative results of non–responders. Out of the total of 62 non–responders we interviewed, 43 (68%) recalled that they received a text message from us. Of those 43 non–responders, 27 (63%) said to have received a reminder text message. A total of 31 non–responders did not know or were not sure whether they received a text message or reminder text message and we asked them for the main reason; most frequently mentioned was do not know (10; 32%), a broken mobile phone (4; 13%), or not checking the mobile phone (4; 13%). The main reasons of the 43 participants for not replying to text messages were as follows: did not have time (13; 30%), did not bring the mobile phone (7; 16%), or mobile phone was switched off (6; 15%).

**Table 1:** Non–responders’ experiences with text messaging survey and reasons for not responding

	No. (%)
**Received text message? (n = 62)**	
Yes	43 (69)
No	11 (18)
Do not know	8 (13)
**Received reminder? (n = 43; “yes” for “received text message?”)**	
Yes	27 (63)
No	7 (16)
Do not know	5 (12)
Missing (interviewer forgot to ask)	4 (9)
**Reasons for not receiving text message (n = 31; “no” or “do not know” for received text message or reminder)**	
Do not know (not related to their mobile phone)	10 (32)
Broken mobile phone	4 (13)
Did not check mobile	4 (13)
Software to block messages	3 (10)
Forgot what happened	3 (10)
Did not bring mobile	1 (3)
Text message box was full	1 (3)
Child played with mobile	1 (3)
Father used mobile	1 (3)
Missing (interviewer forgot to ask)	3 (10)
**Reasons for not responding to text message question (n = 43)**	
Did not have time	13 (30)
Did not bring the mobile phone	7 (16)
Mobile phone switched off	6 (15)
Did not know how to reply	5 (12)
Did not trust the text message	3 (7)
Did not see the text message	3 (7)
Did not have enough credit	3 (7)
Forgot to reply	1 (2)
Child deleted text message	1 (2)
Did not receive a new text message	1 (2)

**Table 2** presents positive and negative views of non–responders on factors influencing follow–up of the text message survey. We found the following factors that had only negative views: mobile phone usage, ability to use the mobile phone, problems with the mobile phone, checking the mobile phone, available time, subjective norm, culture, trust, perceived usefulness of process, and attitude. There were both positive and negative views on perceived usefulness of outcome and ease of use. There were only positive views for actual use (it was mentioned to have replied, but it was too late to reply or we may not have sent a follow–up text message by a mistake).

**Table 2:** Non–responders’ positive and negative views on text messaging survey

Factor	Positive	Negative
Mobile phone usage		Do not send text messages very often
		Not used to sending text messages
		Do not have the habit of replying to text messages
Ability to use mobile phone		Cannot use mobile phone very well
		Cannot reply to text messages
Problems with mobile phone		Mobile phone was broken
		Did not have battery
		Did not have mobile phone credit
		Mobile phone signal is bad
		Text message box full
		Sending the text message failed
		Software to block text messages
		Child deleted text message, could not find it
Checking mobile phone		Did not check the mobile phone
		Did not pay attention
		Was asleep when receiving text message
		Was too late when seeing text message
		Did not see the text message
		Did not bring the mobile phone
		Did not have a ringtone for text message
Available time		Busy, do not have time
		Child was very naughty
		Had something to do at that time
Subjective norm		The child's father did not let mother reply
		Child was playing with mobile phone and father was not at home
Culture		Child’s father used mobile phone
		In “sitting month”*
Trust		Did not trust it
		Did not trust it; there were irrelevant questions in face–to–face interview
		Thought that the phone number should be from Beijing, but text message said “Zhao County Maternal and Child Health Hospital”^†^
		Phone number was too long
		Thought it was a “trash” text message
		Worried about charging fees for text messaging
Perceived usefulness of process		A limitation of the method is that no questions can be asked
		It said to send reminders about raising a child, but these were not send to me
		It took a lot of time to reply
		The face–to–face and text message questions were the same
Perceived usefulness of outcome	It is important	Did not think it was important, because the child did not have the condition that was asked
	It is very good	Did not know why text messages had to be sent
	Good for child’s health	Did not understand why diarrhoea and pneumonia
		Did not matter whether reply was given or not
	Want to make contribution to society	Could not benefit from it directly
Perceived ease of use	Can talk in detail	It was too much effort to reply
	Can understand text messages	Forgot to reply
Attitude		It is inferior to face–to–face or making phone calls
		Do not want to use text message function for surveys
Behavioural intention to use	Will reply when I see it	Did not really want to participate, but cannot say the reason clearly
	Will reply if I have time	
Actual use	Did reply to text message	
	Replied, but it was late	

*In Chinese culture, “sitting month” in brief or “zuoyuezi”, literally means “sitting the first month after delivery” and restricts women from going out of their home or receiving visits from others.

†We explained caregivers that we were from the Capital Institute of Pediatrics in Beijing.

The previously mentioned reasons for not receiving the text message or not responding were also mentioned when we asked further in–depth. While we selected participants based on their ability to text message, some said they could not reply to text messages. Additionally, non–responders said not to send text messages very often. Many reasons for not responding were related to having problems with the mobile phone or not checking the mobile phone. Some non–responders were too busy to respond, especially when the child was naughty. A mother said that the father did not let her reply or that he used her mobile phone. Another reason was being in “the sitting month”; in China traditionally women stay at home in the first month after delivery and have no contact with people outside the family. Trust was a frequently mentioned issue; the text messages were not trusted when the phone number was unusual or when we asked irrelevant questions in the face–to–face interview. Text messaging was perceived as not useful, because no questions could be asked and it took a lot of time to reply. The usefulness of the outcome of the study was perceived important and good for child health. However, some perceived the study not important when the aim of the study was not well understood. Not many views were related to perceived ease of use. Non–responders’ attitude included not wanting to use the text message function for surveys and that it was less good than a face–to–face interview or phone call interview. Positive was that some non–responders had the intention to reply when they saw the text message and had time.

***Non–completers.*** **Table 3** presents quantitative data on non–completers’ views on the surveys. All 58 non–completers recalled to have replied to a text message that they received from us (100%). A total of 36 non–completers (62%) said to have received a reminder message. The most frequently mentioned reasons for not replying were that non–completers replied, but did not receive a new message (34; 59%), did not have time (10; 17%), or forgot to reply (7; 12%).

**Table 3:** Non–completers’ experiences with and views on surveys (n = 58)

	No. (%)
**Received text message?**	
Yes	58 (100)
No	0 (0)
**Received text message reminder?**	
Yes	36 (62)
No	16 (28)
Do not know	5 (9)
Missing (interviewer forgot to ask)	1 (1)
**Reasons for not responding to the text message question**	
Did not receive a new text message	34 (59)
Did not have time	10 (17)
Forgot to reply	7 (12)
Did not have enough credit	3 (5)
Time reading text message was too late	2 (3)
Did not bring the phone	1 (2)
Concerned about privacy	1 (2)
**Views on receiving ¥ 1 for text message costs**	
Was enough	36 (62)
Was not enough	2 (3)
Did not mind	18 (32)
Missing (interviewee quit)	2 (3)
**Preferred study incentive**	
Health information	42 (72)
¥ 5 mobile phone credit	6 (10)
Towel (worth ¥ 5)	4 (7)
No preference	4 (7)
Missing (interviewee quit)	2 (4)
**Preferred survey method**	
Face–to–face	23 (40)
Text messaging	18 (31)
No preference	17 (29)

Qualitative data are presented in **Table 4** on the face–to–face and text messaging survey and in **Table 5** on study incentives. We found the following factors with only negative views: mobile phone usage, ability to use mobile phone, problems with mobile phone, available time, and trust. There were both positive and negative views on checking the mobile phone, study incentives, perceived usefulness of process, perceived usefulness of outcome, perceived ease of use, and attitude. There were only positive views on paying back text message costs and actual use (it was mentioned to have replied, but we may not have sent a follow–up text message by a mistake).

**Table 4:** Non–completers’ positive and negative views on face–to–face and text messaging survey

Factors	Face–to–face survey		Text messaging survey		
	**Positive**	**Negative**	**Positive**	**Negative**	**Other**
Mobile phone usage				Not used to mobile phone	
				Not used to send text messages a lot	
				Used to making phone calls	
Ability to use mobile phone				Mobile phone is not easy to use	
Problems with mobile phone				Mobile phone does not function well	
				Do not have credit	
Checking mobile phone			Take the mobile phone with me	Did not check mobile phone	
Available time		Do not have time for interview		Do not have time; busy, take care of the child	
				Cannot reply timely	
Paying back text message costs			The amount of money is OK		Do not mind about money
			Good to be paid back		It is OK even without; it is for the child, it is honest
					Does not matter; do not have time
					If there were more questions, it is not enough
					The more the better
Study incentive (see **Table 5**)					
Trust		Asked sensitive questions face–to–face		It is hard to trust text messages	Only want to reply to questions about the child
Perceived usefulness of process	Useful because can get information from interviewers			Not useful; cannot get information	
				It is too late, afraid the survey ended, think it is not useful to reply	
		Time–consuming to participate		Time–consuming to reply	
			Faster to reply	Slow to reply	
		Cannot participate in own time	Can reply in own time		
			Can reply when nothing else to do		
Perceived usefulness of outcome			Aim of survey is OK, or good	Do not know the aim of survey	
			It is for the child’s sake	Not useful; child is not ill	
				Only asked question, did not tell how to prevent diseases	
Perceived ease of use		Too much effort to participate, have to go out	Not too much effort	Too much effort to reply	
		Not easy to conduct for researchers	Do not have difficulties, easy to reply	Hard to reply	
				Very likely to make a mistake with a mobile phone	
	Convenient to participate		Convenient to reply	Inconvenient to reply	
				Sending time was not appropriate, was sleeping	
				Will not reply if forget	
				Text messaging scares the child	
	Questions are clear		Questions are clear	Questions are not clear	Questions were the same
	Detailed questions		Detailed questions		
Attitude				Too many text messages	Not so many questions for text messaging, many questions in face–to–face survey
					No preference, methods are equally OK
					Methods are almost the same
Actual use			Did reply to text message		

**Table 5:** Non–completers’ positive and negative views on study incentives

Towel (worth ¥ 5)		¥ 5 mobile phone credit		Health information		
**Positive**	**Negative**	**Positive**	**Negative**	**Positive**	**Negative**	**Other**
Useful	Less useful	More useful	Small amount of credit, is useless	Useful	Will ask village doctor for health problem	Is OK if it is caring about the child, or beneficial
Practical	Can only use the towel once	More practical	Afraid to not receive credit	Cannot get health information		Do not lack these things
Use more towels with a child	Can buy towels	Inconvenient to buy credit in village	Can buy credit	Can use health information for a long time		All the same
	Did not like colour of towel	Use credit more		Health information text messages do not need reply		Will cooperate, no matter what the gift is
	Not worth a lot			On paper is convenient		Do not mind about gift
				You researchers know more than caregivers		
				Important		
				Need to know		

Non–completers were sometimes not used to the mobile phone or not used to sending text messages. In addition, they experienced some problems with their mobile phone. They did not always check their mobile phone, but could reply when they brought their mobile phone in their pocket. Both for the face–to–face and text messaging survey there were non–completers who said not to have time. Paying back ¥ 1 for the text messages was enough for 36 out of 58 non–completers (62%). Non–completers said that paying back the text message costs was good, but also mentioned not to mind about the money, because it was for the child’s sake. However, more money was found to be better and if there were more questions, ¥ 1 would not be enough. As incentive, 42 out of 58 participants liked to receive health information (72%). Health information was found useful and important, because it could be used for a long time and there was a need for more information. In addition, health information was harder to obtain than the towel that we gave for the face–to–face interview or the ¥ 5 mobile phone credit (which we promised to give if non–completers responded to all text message questions). However, non–completers also mentioned that they did not mind about the incentives and did not lack them. Non–completers found it hard to trust the text messages and concerns about privacy were raised, because we asked sensitive questions (about income and expenditure) in the face–to–face survey. Some non–completers only wanted to reply to questions about the child.

There were many comments related to perceived usefulness and ease of use. The face–to–face survey was perceived to be more useful than the text messaging survey, because questions could only be asked during the face–to–face interview. Both methods were found time–consuming. However, a perceived benefit of the text messaging method was being able to respond at a self–chosen time. While some did not know what the aim of the text messaging survey was, others perceived the aim to be OK or good. It was found too much effort to participate in the face–to–face survey, because it required going out. There were some contradicting views on ease of use. For example, replying via text messaging was found both not much effort and too much effort, both easy and hard, and both convenient and inconvenient. The questions were found to be clear and detailed for both methods, but text message questions were also found to be unclear and mistakes were likely to happen via text messaging. Out of the 58 non–completers, 23 (40%) preferred the face–to–face survey method and 18 (31%) the text messaging method. When asked in–depth, many non–completers expressed to have no preference and that the methods were equally OK. However, non–completers also said that there were too many text messages.

***Completers.*** **Table 6** presents the quantitative results of completers. Of the total of 73 completers we interviewed, 26 (36%) said to have received a text message reminder and the majority of them (24; 92%) found receiving one, two or three reminders OK. Nevertheless, still some completers worried about forgetting to reply. The time of receiving the text messages was acceptable for 48 participants (66%). The evening or afternoon was the most preferred time to receive text messages and 15 said that any time was OK (21%).

**Table 6:** Completers’ experiences with and views on surveys (n = 73)

	No. (%)
**Received text message reminder?**	
Yes	26 (36)
No	41 (56)
Do not know	1 (1)
Missing (interviewee quit)	3 (4)
Missing (interviewer forgot to ask)	2 (3)
**Acceptability text message reminder (n = 26)**	
Received 1 reminder; is OK	15 (58)
Received 2 reminders; is OK	7 (27)
Received 2 reminders; is too much	1 (4)
Received 3 reminders; is OK	2 (7)
Missing (interviewer forgot to ask)	1 (4)
**Time receiving text message acceptable?**	
Yes	48 (66)
No	22 (30)
Do not know	1 (1)
Missing (interviewee quit)	2 (3)
**Preferred time for text message survey**	
Morning	4 (6)
Morning or afternoon	5 (7)
Morning or evening	2 (3)
Afternoon	16 (22)
Afternoon or evening	11 (14)
Evening	17 (23)
Any time	15 (21)
Do not know	1 (1)
Missing (interviewee quit)	2 (3)
**Views on receiving ¥ 1 for text message costs**	
Was enough	63 (86)
Was not enough	1 (1)
Did not mind	5 (7)
Missing (interviewee quit)	3 (5)
Missing (interviewer forgot to ask)	1 (1)
**Views on receiving** ¥ **5 mobile phone credit for completing text message survey**	
Was enough	60 (82)
Was not enough	1 (1)
Was too much	2 (3)
Did not mind	6 (9)
Do not know	1 (1)
Missing (interviewee quit)	3 (4)
**Preferred incentive**	
¥ 5 mobile phone credit	35 (48)
Towel (worth ¥ 5)	6 (8)
Health information	16 (22)
No preference	13 (18)
Missing (interviewee quit)	3 (4)
**Number of text message questions willing to answer on one day?**	
1–2	4 (6)
3–4	14 (19)
5–6	23 (32)
7–8	1 (1)
>8	20 (27)
All OK	5 (7)
Missing (interviewee quit)	6 (8)
**Number of text message questions willing to answer in total?**	
3–4	1 (1)
5–6	3 (5)
7–8	1 (1)
>8	56 (77)
All OK	5 (7)
Missing (interviewee quit)	6 (8)
Missing (interviewer forgot to record)	1 (1)
**How often willing to respond to text message survey?**	
Once a month or more often	51 (70)
Once every 2 months	4 (6)
Once every 3 months	6 (8)
Once every 6 months	2 (3)
All OK	1 (1)
Do not know	3 (4)
Missing (interviewee quit)	6 (8)
**Preferred survey method**	
Face–to–face	35 (48)
Text messaging	35 (48)
Phone call	1 (1)
No preference	2 (3)

Qualitative data are presented in **Table 7** on the face–to–face and text messaging survey and in **Table 8** on study incentives. We found the following factors with only negative views: mobile phone usage, ability to use the mobile phone, and checking the mobile phone. There were both positive and negative comments for available time, study incentives, trust, perceived usefulness of process, perceived usefulness of outcome, perceived ease of use, and attitude. There were only positive views on problems with the mobile phone and paying back text message costs.

**Table 7:** Completers’ positive and negative views on face–to–face and text messaging survey

Factors	Face–to–face survey		Text messaging survey		
	**Positive**	**Negative**	**Positive**	**Negative**	**Other**
Mobile phone usage				Do not use text messaging very often	
Ability to use mobile phone				Not easy to communicate via text messaging	
Problems with mobile phone			Have enough credit		
Checking mobile phone				Sometimes will not reply immediately, because do not see text message	
				Afraid that I cannot receive the text messages	
				I am unsure whether I can see the text message	
Available time	Have time for interview	Do not have time for interview	Will reply in spare time	Normally do not have time	
				Do not have time to send text messages	
Paying back text message costs			Ok or good to be paid back		Do not know
			Practical		Do not mind
			Do not mind about that, but better to be paid back		
			Did not need to send many text messages, does not cost a lot, not enough when there are more text messages		
			Good that parents do not have to pay		
			Depends on the aim, it is for the child, so does not matter		
			Do not have to pay for it		
Study incentive (see **Table 8**)					
Trust			Replied because you first contacted me face–to–face	Text messages are hard to trust	
			Gave honest replies	There are a lot of cheating text messages	
				Cannot reply as you required, afraid that replying in format results in higher costs	
				Do not reply to a strange number	
Perceived usefulness of process	Not time– consuming	Time–consuming	Faster than face–to–face interview	Takes a long time to reply	
	Can ask questions directly			Inconvenient for me to ask questions	You can easily ask questions in phone calls
			Saves time		Phone calls save time
			Saves time, can continue work	Cannot reply at work	Phone calls take a long time
			Can reply in own time when not busy		
	Convenient		Convenient to reply	Inconvenient to reply	Phone calls are convenient
		Have to be at the clinic at a set time	Do not have to be at a particular place	Inconvenient for grandparents to reply	
			Do not need to go out	Inconvenient to reply when taking care of child	
			No time limit for replying to text messages		
	More detailed		Questions are detailed	Text messages are not detailed	
			You can get specific information; you asked a lot of questions	It is too simple, you only asked a few questions	
Perceived usefulness of outcome			Aim of survey is OK, good, or very good	Aim of your work is not so useful	You asked the same questions face–to–face and via text messaging
			You care about the child		
			You can follow the child's health condition	Child does not have symptoms you asked	Worth replying if I can ask questions
			You can understand my child’s health condition immediately		
			It makes me conscious about my child's health condition		
Perceived ease of use	Easy	Too much effort	Simple	Too much effort	
				Will not always reply	
				It is easy to forget to reply	
			Good to have a long interval between text messages	Text message software is slow, too long interval between text messages	You can explain things in phone calls
			Do not need to talk		
				When having questions, will not ask	
	Clear		Text messages are clear	Text messages are not clear	Phone calls are clear
			Can understand the text messages	Not easy to understand the text messages	
			Content of text messages is good/ questions are good	Did not understand the question	
	Feel good because it is intimate	Feel embarrassed	Feel at ease	Bothering	
			Easy to recall	Some of the text messages were repeated	
			Have time to think about it	There are things that you cannot say in text messages (complicated things)	
			Better to read than listen	Will be distracted when sending text messages	
Attitude			Text messaging method is good	Text messaging is less good than making phone calls	All methods are OK
			Way to do it is good	Text messages were too frequent	Like both methods
			Want to cooperate with your work	Get annoyed when receiving many text messages	Both methods have their own benefits
			Want to make contribution to society		Methods are (almost) the same, no preference
			Willing to participate		

**Table 8:** Completers’ positive and negative views on study incentives

Towel (worth ¥ 5)		¥ 5 mobile phone credit			Health information		
**Positive**	**Negative**	**Positive**	**Negative**	**Other**	**Positive**	**Negative**	**Other**
		It is OK, or good		It depends, it is hard to say	Need health information		All of them are OK
		Nice gift, shows that you are kind	Makes me feel that it has other bad purposes	Does not matter, will reply anyway	Hope to know more about child health		
Child likes gift		Nice surprise		It is for the child	Child health is important		It is OK to get it or not, if it is for the child
							Want the child to be happy
Useful		Useful					
		Convenient					
	Easy to get gift, not precious	Good you recharge credit, it is inconvenient to recharge					
Benefit	Worried about quality of gift	Benefit				2012 calendar is not good*	
	Is not worth much	It is a lot, not necessary	Reward is not a lot	Small amount of money, do not mind			
	Do not want a small gift	Would like to reply when getting ¥ 5	It only paid back the credit I used	The more the better			
		It is an incentive, makes it more likely that I reply					
		Good that parents do not have to pay for text messages		If there are many participants, it is a lot of money			
		Good to be paid, because it takes some of my work time to reply					
		Money for the time I spent					
		Feel that I did not do so many things					

*We gave a calendar with infant feeding information, which we developed in a previous study in 2012.

Although completers replied to all text message questions, limited text messaging usage and ability were seen as hindering factors when responding to text messages. Completers did not mention problems and had enough mobile phone credit to respond. Completers were not being able to reply immediately when they did not see the text message. Both for the face–to–face and text messaging survey there were completers who said to not have time for the interviews. It was hard to trust the text messages, because there were a lot of text messages that were perceived as deceiving, and thus no reply would be given to a strange number. However, having first face–to–face contact encouraged completers to reply and honest replies were given. A total of 63 out of 73 completers (86%) found being paid back ¥ 1 for their text message costs enough. This was also found good and practical. Some said that they did not have to send so many text messages and that sending text messages did not cost a lot. The incentive of ¥ 5 was found enough by 60 completers (82%) and this was the preferred incentive for 35 completers (48%). The most comments about incentives were related to the positive aspects of receiving ¥ 5 credit; some said that they did not expect the credit and that it was a nice surprise. Also, credit was found convenient, because it was not easy to buy the credit in the villages. However, some felt the amount was too much and that their effort was not enough for receiving ¥ 5. Negative comments included that it was felt that the survey may have other purposes when ¥ 5 was given. Only 16 completers (22%) preferred health information. Health information was valued, because it was important and needed. However, the received calendar with infant feeding information was from last year (2012) and hence less useful. Some completers said that the child liked the towel that we gave for the face–to–face interview, while others found it not worth much and did not want a small gift.

There were many comments related to perceived usefulness and ease of use and many of them were contradictory. Both methods were perceived to not take much time, but also to be time–consuming. For the text messaging method, it was valued that a reply could be given at a self–chosen time and not having to be at a certain place. However, the text messaging survey was found less useful, because no questions could be asked. Both methods were found convenient, but it was inconvenient to reply to text messages, especially for grandparents. The aim of the text messaging survey was perceived as OK or good, because the child’s health condition could be followed and we showed that we cared about the child. However, it was not perceived useful when the child did not have the disease symptoms we asked about. Both the face–to–face and text message were perceived as easy, but also too much effort. The text messages were found to be clear, detailed and understandable, but also unclear. For the face–to–face interview, feeling good was mentioned, but also feeling embarrassed. For the text message survey, completers said to feel at ease, but also to feel bothered.

The text message method was found to be good. Completers wanted to cooperate with our work and make a contribution to society. Most completers were willing to receive at least 3–4 or more text messages a day (63; 79%), and more than eight text message questions in total (61; 84%). However, it was also mentioned to get annoyed when receiving too many text messages. A total of 51 participants (70%) said to be willing to complete a text message survey at least once a month. Frequently mentioned was that all methods were OK and to not have a preference. However, when completers had to choose, 35 (48%) preferred the face–to–face and 35 (48%) the text message method.

***Researchers.*** We found four additional factors influencing follow–up: (i) different caregivers participating in face–to–face and text message survey, (ii) sending text messages manually, (iii) caregivers’ understanding of survey questions, and (iv) technical issues.

First, we found that 93 caregivers who came to the village clinics for the face–to–face survey were not the same person participating in the text messaging survey (**Table 9**): 8 participants in group 1 (of whom 6 replied the first survey text message question) and 85 participants in group 2 (of whom 76 replied to the first survey text message question and then participated in the face–to–face survey). This was mostly because mothers (46; 49%) replied to the text messages, but fathers (10), grandmothers (26), grandfathers (9) or another person (1) came to the face–to–face interview. Also frequently happened that the father responded to the text messages (40; 43%), but mothers (25), grandmothers (11) or grandfathers (4) came to the face–to–face interview. These caregivers had to be excluded from the analyses. In group 1, of the 189 participants who responded to the first survey text message question 183 caregivers (97%) were the same caregivers in the face–to–face and text message survey. In group 2, of the 302 participants who completed the face–to–face interview and responded to the first survey text message question, 226 (75%) were the same caregiver who participated in the text message and face–to–face survey.

**Table 9:** Number of different caregivers participating in face–to–face and text messaging survey (n = 93)

Face–to–face	Text messaging				
	**Mother**	**Father**	**Grandmother**	**Grandfather**	**Other caregiver**
**Mother**	0	25	2	1	3
**Father**	10	0	0	0	1
**Grandmother**	26	11	0	0	0
**Grandfather**	9	4	0	0	0
**Other caregiver**	1	0	0	0	0
**Total**	46	40	2	1	4

Second, sending text messages manually was a labour intensive process. One researcher (YL) was continuously sending text messages 12 hours a day (from 9 am till 9 pm) during the study period (14 days). Before and after sending text messages, she had to do additional work for the study (communicate with the fieldworkers and preparation and checking work). Sending text messages was relatively complicated, because we had to send text messages at different times to the two groups and we had to send participants questions depending on the response they gave. Therefore, we could not find a suitable Chinese automated text messaging system for this study. This manual work was prone to errors and a second person checked the text messages that were sent out. The researcher sent files containing ten messages to the second person who checked them. About one in five files contained one or two mistakes, which had to be revised. Even after these checks errors occurred, which was confirmed by participants who said that they did not receive follow–up text messages and therefore could not complete the survey. We checked this and found that indeed we did not sent text messages to some participants. We assessed that about two percent of the messages were incorrectly sent (unpublished).

Third, despite carefully designing our text message survey [28], questions were not always understood or participants did not reply in our requested format. We found that about one in three text message responses were in an incorrect format and had to be checked. However, only a small proportion (about 4%) of participants had to be sent the text message again, which was mainly for questions about where care was sought (unpublished). This resulted in delayed follow–up of participants.

Fourthly, a positive factor for follow–up was that we did not experience technical issues such as network problems or issues with our text messaging system during the study.

**Recommendations for improving follow–up.** **Table 10** presents non–responders’, non–completers’, and completers’ recommendations for improving follow–up and our views. Non–completers provided more recommendations than non–responders and completers. A number of participants said to not know what could be changed to improve follow–up. This was just the situation in rural areas in China, by which was meant that now more parents had to go to work and grandparents then took care of the child. It would be better to do the research at a place where more parents took care of the child. There were no good solutions for changing the text message survey, because caregivers could not take the mobile phone with them all the time or would forget to bring it, some parents and many grandparents could not text message, and the text messaging method depends on the initiative of caregivers.

**Table 10:** Participants’ and our recommendations for improving follow–up

Participants’ recommendations		Our recommendations
Non–responders	Explain purpose more clearly	Develop and test new information materials
	Should pay attention to hand, foot, mouth disease*	Explore sending participants health information of their interest
	Using a normal mobile phone number to send text messages	Technically not feasible; instead informing participants about the phone number
	Should mention “Capital Institute of Pediatrics” in text message	Feasible
	Send text message at an appropriate time	Explore giving participants the option of choosing a time of their convenience at which text messages are sent
	It is convenient to make phone calls	Not feasible, too time–consuming and costly
	Do not know/ Do not have comments	–
Non–completers	Explain the aim more clearly	Develop and test new information materials
	Inform in different ways, village doctors, advertisement and so on	Explore different ways of informing caregivers
	Increase trust: use familiar number, sending a greeting, providing consultation	Explore these ways to increase trust
	Need to tell what to do with symptoms	Explore sending text messaging with health information of interest
	Send feedback	Explore sending text messages with feedback
	Pay for text messages immediately	Was technically not feasible; explore having a free text message number
	Send text messages at an appropriate time	Explore giving participants the option of choosing a time of their convenience at which text messages are sent
	Hope (the investigator) can send text messages quicker	Was technically not feasible; explore option
	Send all questions in one text message	Not feasible, does not fit in one text message and sending all text messages simultaneously was also not possible because questions depended on answers
	Should not be so many text message questions	
	Send more text message reminders	Explore giving participants the option of choosing how many reminders are sent
	Send fewer text message reminders	
	Using text messaging for follow–up study	Good strategy
	Ask questions by making phone calls	Not feasible, too time–consuming and costly
	Not so much needs to be changed	–
	Do not know	–
Completers	Focus on more common diseases	Explore sending text messaging with health information of interest
	Hope you can give consultation about child’s health	Explore sending text messages with feedback
	Need feedback for the text messages sent	
	Do not have comments	–

*Infectious child disease, usually caused by Coxsackie virus. Symptoms are blisters on hands, feet and mouth, and fever.

## DISCUSSION

### Principal results

**Recruitment.** Of the 4170 names of caregivers of children under five, we recruited only 1026 (25%) and included 1014 (24%) caregivers. Based on interviews with village doctors and our experiences, we found several factors explaining this finding and recommendations for ways to recruit more caregivers.

***Factors influencing recruitment.*** We found the following factors influencing recruitment: reachability of villages, fieldwork schedule, availability of village doctors, efforts of village doctors, availability of name list, availability of phone numbers, time of recruitment, selection criteria for recruiting caregivers, using the villages’ loudspeaker, making phone calls visiting caregivers’ houses, and other recruitment methods. Furthermore, village doctors’ work–related factors were their duties, division of their work, work load, gathering caregivers for vaccination, and experience with recruiting caregivers for previous studies. Village doctors’ motivations were their understanding of the study, interference with work, money, work for villagers, follow orders from township and county hospital doctors, and cooperate with research team. Factors related to caregivers’ characteristics were their education and ability to text message. Caregivers’ motivations included understanding of the study, interference with work, trust, sensitive questions, reward (towel), and health information.

***Recommendations for ways to recruit more caregivers.*** Feasible ways to recruit more caregivers were giving the name list and phone numbers in advance, visit villages earlier on the day, continue using the villages’ loudspeakers, continue making phone calls and send text messages, give village doctors more time for visiting caregivers’ homes, and bring a doctor for free consultation and send health information text messages. Moreover, to address factors that negatively influenced recruitment, we suggest developing and testing new information materials for village doctors and caregivers, omitting sensitive questions from survey, and tailoring recruitment strategies to the specific context of villages.

**Follow–up.** Of the 1014 participants included in the cross–over study, 662 (65%) responded to the first question about willingness to participate, 538 (53%) responded to the first survey question, and 356 (35%) completed the text message survey. Of the 349 participants in group 2 who were required to return to the village clinic for the face–to–face interview, 302 (87%) attended. Based on the interviews with participants and our experiences, we found several factors explaining these findings and recommendations to improve follow–up.

***Factors influencing follow–up.*** In interviews with non–responders, non–completers and completers, there were mainly negative views on factors influencing follow–up related to mobile phone use, ability to use the mobile phone, problems with the mobile phone, checking the mobile phone, available time, trust and culture. Participants’ limited mobile phone use or inability to use the mobile phone to text message restricted them in replying to text messages. Non–responders seemed to have more problems with their mobile phone and with checking their mobile phone than non–completers and completers. Participants mentioned not having time for both the face–to–face and text message surveys. Non–completers and completers perceived paying back text message costs as a positive factor and had varying views on the incentives. Non–completers seemed to have been keener to participate when health information was provided, while completers seemed happy with the ¥ 5 reward for completing the survey (which non–completers did not receive). Non–responders only had negative views on perceived usefulness of the process, while non–completers and completers also had positive views. All different participants had both positive and negative views on perceived usefulness of the outcome and perceived ease of use.

We found four additional factors influencing follow–up: different caregivers participating in face–to–face and text message survey, sending text messages manually, caregivers’ understanding of survey questions, and technical issues. We found that mainly when mothers responded to the text message survey, grandmothers participated in the face–to–face survey, or that fathers responded to the text message survey and mothers participated in the face–to–face survey. Sending text messages manually was time–consuming and introduced errors. Also, despite carefully designing the text message survey, errors occurred when caregivers did not understand the questions. Positive was that we did not experience technical problems during the study.

***Recommendations for improving follow–up.*** Recommendations to improve follow–up were various. Based on participants’ recommendations, we suggested a number of strategies to improve follow–up including the following: developing and testing new information materials, sending health information and feedback text messages, explore ways to increase trust and tailoring the text message survey to participants’ preferences.

### Strengths and limitations

To our knowledge, this is the first study exploring factors influencing recruitment and follow–up in an mHealth study in a middle–income country. The study took place in rural Northern China and our pragmatic approach provided information about a real–life setting. We have been conducting studies for a number of years in our field site and we had good relations with the local health workers. Therefore, we were aware of the local customs and could communicate in the local dialect (which is slightly different from standard Chinese) [28].

We provided an overall perspective by evaluating views from both village doctors and participants of the cross–over study, and by adding our own views. To ensure that the interpretation of the meaning of the data was correct, we collected and analysed the data in Chinese, translated the main findings into English, translated the English main findings back into Chinese, compared this with the original data and resolved disagreements. In addition, we had several discussions in our research team to confirm validity of findings. However, with any translation some meaning of the original language will be lost.

Our study was exploratory and only provided insights in factors that influenced recruitment and follow–up. We could not assess the effects of strategies used to improve recruitment and follow–up. Also, we could not collect detailed information on non–consenters and thus we could not assess the amount of selection bias.

The interviews covered a broad range of issues and we did not reach saturation on specific issues. Participants provided critical and interesting insights in this underexplored research area. However, the findings need to be interpreted with caution, because we felt that participants sometimes gave socially desirable answers to our questions. We put these findings into perspective by adding our views. While the sample of village doctors was relatively small (N = 10), we interviewed village doctors from different villages, both male and female, aged between 29 and 63, and village doctors who had their own clinic and village doctors who shared a clinic. Despite our random selection, there were no grandmothers or grandfathers and in the sample of non–responders of the cross–over study. Therefore, views of those participants mainly represent parents’ views.

Telephone interviews have their own benefits and shortcomings [43,44]. We were able to interview a relatively large number of participants in a short amount of time without having to revisit all the villages, which practically would have been very difficult. Nevertheless, multiple methods of communication that are used in face–to–face interviews (body language and other visual cues) could not be used to interpret and communicate with the participants in telephone interviews. In addition, interviews were not audio taped and we relied on written responses of the interviewers.

### Comparison with prior work

We conducted two mHealth data collection studies on infant feeding in our setting [45,46]. The first study (N = 258) took place a year before the cross–over study and aimed to evaluate the use of text messaging for program monitoring [45]. Based on our findings in this study, we checked the mobile phone number of caregivers ourselves (instead of asking village doctors to check them), we sent two reminders instead of one and we took into consideration the time at which most caregivers responded. In addition, we asked interviewers to remind caregivers to reply, to explain the format in which they had to reply and told them that they would receive ¥ 5 credit if they replied to all text messages. The second study (N = 591) is reported in this mHealth series and took place four months after the cross–over study [46]. The study explored the feasibility of text messaging data collection of infant and young child feeding practices. In that study, we sent all text message questions simultaneously to participants. We were unable to do this in the cross–over study, because the questions that we sent depended on the answer participants gave.

Representativeness of mHealth study samples is an important issue for selection bias. In our setting, the illiteracy rate was low and we did not find problems with illiteracy in current and previous research [45,46]. Mobile phone use was high and most parents could text message, but many grandparents could not text message and thus had to be excluded from participation. However, this may not be problem in settings where elderly people are able to text message [47]. Previous mHealth data collection studies frequently only included younger participants [10–13,15,20,21]. While in other settings socio–economic factors may influence use of mobile phones and text messaging [9], this did not seem to be of large influence in our setting as the costs of text messaging were very low (receiving text messages is free and sending one text message costs ¥ 0.1, about £ 0.01, € 0.01, US$ 0.02).

When participants are recruited in mHealth studies, it is important to know whether participants who are followed up are different to those who are lost to follow–up. In our second mHealth study, we did not find significant differences in demographic characteristics between responders and non–responders of the text message survey in our second mHealth study [46], and a similar finding was reported by an mHealth data collection study in Sweden [19]. Retention bias is influenced by a number of factors. Text messaging is more likely to work when there is follow–up, the text message is personally tailored and the content and frequency are highly relevant [1]. Researchers have successfully followed participants up in mHealth data collection studies by having face–to–face contact, sending text message reminders, making phone calls, and sending letters [11,15,19]. In our study, having face–to–face contact with caregivers seemed effective in increasing caregivers’ trust, but we found that it remained hard to gain caregivers’ trust. Not surprisingly, participants mentioned to not trust the text messages in the interviews. We made phone calls and sent text messages to participants in group 2 to ask them to go to the village clinic for the face–to–face interview and achieved a high return of participants (87%). We sent two text message reminders and participants seemed to find this acceptable. Some participants perceived the text messages a reminder for their child health. A study in Kenya found that data collection text message served as medication reminders [10].

Some of the reasons for not responding to text messages we found have been previously reported. In our first mHealth study, we found similar reasons for not replying, including not receiving text messages, being too busy to reply, or not seeing the text message on time [45], but in the current paper we were able to give more in–depth explanations for reasons. As in our study, researchers in Thailand did not pay for the text messages and participants’ ran out of credit. Also, participants mentioned running out of battery, technical challenges and not keeping mobile phones with them all the time [15]. A study in Uganda found that poor understanding and fear of making mistakes was an important challenge for completion of text message data forms [10]. We also found that some participants said it was likely to make mistakes via text messaging.

In our other mHealth studies, we used a smartphone to send the text messages [45,46], while we sent text messages manually with a text message system in this study. Manually sending text messages introduced errors, but this would not be completely resolved with an automated text message system. A study in the UK used an automated text messaging system, but out of 2952 text messages sent in total, still 214 (7%) text messages had to be sent manually [11]. As a result of system or researcher errors, about 6% of participants were sent the wrong number of text messages (too many or too few), while for about 2% of participants there were other problems [11].

The use of appropriate theory is often lacking in mHealth studies [1]. Although the Technology Acceptance Model was not specifically developed for the health care context, it has been used by a large number of studies for health care and is increasingly seen as fitting [48]. The model has mainly been used for predicting and explaining health workers’ acceptance and use of health care information technology [49], but has also shown predictive value for consumers’ adoption of health care information technology [50–52]. The model predicts a substantial portion of the use or acceptance of health care information technology, but may benefit from several additions and modifications. It has been recommended to further contextualize the Technology Acceptance Model to health care, which can uncover the specific meaning of generic variables [48]. A small number of mHealth studies have used the Technology Acceptance Model [49–51]. We found that the Technology Acceptance Model provided a useful framework for understanding follow–up of participants in our study.

### Future research

There are a number of questions that remain and require further research. In our setting, the accuracy of the name list needs to be assessed by reporting how many children on the name list cannot be found in the villages. In addition, it needs to be reported how many caregivers are not able to participate and for what reasons. The suggested strategies to improve recruitment and follow–up need to be tested and their effectiveness needs to be assessed. The use of an automated text message system needs to be explored to reduce the work load of researchers and to further improve accuracy of sending the text messages. This would also allow us to send text messages quicker and at participants’ preferred times. Future studies could use the Technology Acceptance Model to develop interview guides [53] and test the variables in the model for mHealth data collection [51].

## CONCLUSIONS

This is the first study to explore factors influencing recruitment and follow–up of participants in an mHealth data collection study in a middle–income setting. The lessons learned in this study emphasize the importance of rigorously testing mHealth interventions in a new setting. More work is needed to implement our suggested strategies and assess their effectiveness. This work would be valuable as there is currently limited information available that can guide sample size calculations for mHealth–based studies. Knowing more about recruitment and retention of participants in mHealth studies would be an important step in improving mHealth evaluation. When mHealth interventions are sufficiently evaluated, successful mHealth interventions could be scaled–up and ultimately support the delivery of health care and improve health [1].
