# Supplementary material for: mHealth Series: Factors influencing sample size calculations for mHealth–based studies – A mixed methods study in rural China
Source: J Glob Health. 2013 Dec;3(2):020404. doi: 10.7189/jogh.03.020404 (PMC3868817; doi:10.7189/jogh.03.020404)
Supplement: Online Supplementary Document [file jogh-03-020404-s001.pdf]

Online Supplementary Document

van Velthoven et al. mHealth series paper 4: Factors influencing sample size calculations for mHealth-based studies - A mixed methods study in rural China.
Journal of Global Health 2013;3:020404

Overview of detailed results for recruitment

We provide a detailed description of village doctors' and researchers' views on recruitment:

i. Factors influencing recruitment of caregivers (Table S1);

ii. Recommendations for ways to recruit more caregivers (Table S2).

We identify villages and interviewed village doctors (I) by their number as shown in Table S3 for group 1 and Table S4 for group 2 at the end of this Online Supplementary Document.

Factors influencing recruitment of caregivers

Table S1 shows an overview of themes and related factors influencing recruitment of caregivers that we found in the interviews with village doctors and based on our views. In the following sections, we describe each theme and related factors in detail.

Table S1. Themes and related factors influencing recruitment of caregivers

Themes	Factors influencing recruitment
Experiences with recruitment	Reachability of villages<br>Fieldwork schedule<br>Availability of village doctors<br>Efforts of village doctors<br>Availability of name list<br>Availability of phone numbers<br>Time of recruitment<br>Selection criteria for recruiting caregivers<br>Using the villages’ loudspeaker<br>Making phone calls<br>Visiting caregivers’ houses<br>Other recruitment methods
Village doctors’ work	Duties<br>Division of work<br>Work load<br>Gathering caregivers for vaccination<br>Recruiting caregivers for previous studies
Village doctors’ motivations	Understanding of study<br>Interference with work<br>Money<br>Work for villagers<br>Follow orders from township and county hospital doctors<br>Cooperate with research team
Caregivers’ characteristics	Education<br>Ability to text message
Caregivers’ motivations	Understanding of study<br>Interference with work<br>Trust<br>Sensitive questions<br>Reward (towel)<br>Health information

Experiences with recruitment. We found the following factors influencing recruitment: (i) reachability of villages, (ii) fieldwork schedule, (iii) availability of village doctors, (iv) efforts of village doctors, (v) availability of name list, (vi) availability of phone numbers, (vii) time of recruitment, (viii) selection criteria for recruiting caregivers, (ix) using the villages' loudspeaker, (x) making phone calls (xi) visiting caregivers' houses, and (xii) other recruitment methods. We present our experiences with recruitment in group 1 in Table S3 and in group 2 in Table S4 at the end of this Online Supplementary Document.

Reachability of villages. Generally, we had no problems with reaching villages. However, one village in group 1 could only be visited once (village 27), because of road works. During the first visit, we had to walk from the end of the village to the village clinic. When we wanted to visit the village for the second recruitment round, the road was completely blocked and we were unable to enter the village.

Fieldwork schedule. We had to visit villages on set days to have equal intervals between visiting villages and sending text messages. In general, we succeeded to make sufficient time available for recruitment in each village, but it was challenging to reschedule when something went wrong. Two different villages were scheduled at the same time (8.00 am) on the same day, because of a communication mistake. We found out after we finished recruitment of participants in the first village (village 7; finish 9.30 am). When we arrived in the second village (village 8), many caregivers were waiting for us and expressed their dissatisfaction. We had to take much time to explain the situation, and there was no time left to call all caregivers, because we had to visit other villages on our schedule.

Availability of village doctors. Village doctors said that they were available for the study, because it was not busy at the time of the study (March) (I8) and thus we did not delay their work (I5, 6, 7). In villages with more than one village doctor (I4, 5), their work was divided among each other and then it was more likely that someone was available to help us (I4).

We found that while most village doctors were available to gather caregivers, some were not available during certain days; a village doctor was not available in the weekend (I8; village 43). In other villages, the village doctor was not available at all; a village doctor was not available for a personal reason (village 30) and a village doctor was too busy (village 14). The township hospital doctor said that two villages (village 6 and 45) did not have a village doctor.

Efforts of village doctors. Some village doctors (I1, 2) did not clearly say whether they thought that they could have gathered more caregivers. Others found that because the study was voluntary, they did not have to reach everyone and did not try their best to recruit all caregivers (I1, 9). They tried to ask caregivers and if caregivers did not want to participate, then they did not persuade them.

From our perspective a number of village doctors seemed to do everything they could do to participate. It was difficult for village doctors to gather caregivers, especially in small villages with few children. Sometimes the village doctor knew the villagers well, which facilitated recruitment. Nevertheless, some village doctors were not very helpful. We could not recruit any caregivers in one village (village 14), because the village doctor was too busy with work and our efforts to recruit caregivers were not successful. In another village, the county hospital doctor said that he could not reach the village doctor for two days by mobile phone. When the county hospital doctor visited the village, he found the village doctor drunk (I3; village 25). When we arrived in the village, the village doctor did not want to make the announcement with the loudspeaker, then disappeared and could neither be found in the clinic (while the door was open) nor at home. A teacher in the local nursery told us that "the village doctor was not good". When we revisited the village, we found the village doctor and asked him why he was not available. He said to be in the county hospital when we were looking for him during the first visit, but gave the impression to lie during the interview (he was sitting with his arms crossed and his knees were shaking).

Availability of name list. In the first two villages that we visited (I4; village 40, and I9; village 46), we did not give the name list from the township hospital to village doctors, because our previous experience was that they had their own name list. However, village doctors only had a name list of mothers who had been pregnant and received folic acid. Village doctors did not have their own name list of children under five and therefore we provided the name list to all village doctors. Village doctors said that receiving the name list was useful and allowed them to find more caregivers (I2). Nevertheless, the name list seemed inaccurate, because we found children who were not on the name list and village doctors said that not all children on the name list were living in their village (I6).

Availability of phone numbers. While some village doctors provided us caregivers' mobile phone numbers, they usually did not have them or not all of them (I2, 3, 4). When village

doctors had the mobile phone numbers for a longer time, they were not sure whether those numbers were still correct. When we gave phone numbers of caregivers, this helped to find caregivers (I2).

For some of the villages we had a list with phone numbers of caregivers from the township hospital. Many of these phone numbers were incomplete (number replaced by *), because the township hospital could not provide the complete numbers. The available complete numbers were mainly mobile phone numbers, but also several landline telephone numbers. We found that most of the landline telephone numbers were not valid. People preferred to use a mobile phone and therefore did not pay the fee for the telephone, which resulted in the line being shut off.

Time of recruitment. Village doctors thought that the time at which we visited villages (during the day) was not the most appropriate time to recruit caregivers (I2, 7). They said that it was difficult to find mothers and fathers, because parents went out for work (I1, 3, 7, 8, 9). Some parents came back home in the evening (from around 7 pm) (I6), while others stayed at work for several weeks or longer, sometimes in a different county or in a different province (I8).

"VD: As I told you that day, our village is mostly empty. 我那天不是跟你说了，基本上这都是空心村。

WW: Yes. I see. 嗯。嗯 嗯。

VD: Parents are rarely at home. 父母很少在家的。

WW: Ok. 嗯。

VD: Mostly it is the grandparents who stay at home. 大部分都是爷爷奶奶在家。" (I3)

The day in the week on which recruitment took place did not matter, because many caregivers worked on all days of the week. We found that it was more common that parents were not at home in downtown villages than in rural villages.

Selection criteria for recruiting caregivers. Most village doctors thought that the selection criteria limited their ability to recruit caregivers, because not all caregivers could text message.

"VD： The selection criteria were very strict. 这几条就很限制。

DXZ：Many people were not able to participate, right? 就卡掉很多人是么。

VD： Yes. Yes. 对。对。" (I8)

Using village's loudspeaker. We found that some villages had no loudspeakers. Villages that had a loudspeaker either had a private loudspeaker for which had to be paid, or a free loudspeaker. When loudspeakers were available, most village doctors (I1, 2, 3, 5, 10) used them. For a number of interviewed village doctors (I1, I2, I10), this was the only way they tried to recruit caregivers. Some village doctors used the loudspeaker in the morning (I7) and some in the evening (I6) before we arrived in the village. In addition, some village doctors read the names of caregiver on name list one by one when making the announcement (I2, 6, 7).

"VD: I just told parents to come (to the village clinic). You see the name list you gave me, I read it entirely. 就是告诉叫人爸爸妈妈来。 就你给的那个单上的名是吧，统统念了一遍。

WW: Ok. The entire name list was…哦您把整个的那个名单。。。

VD: Yes, I read every name on it. 对，整个……整个那上面的名给全念了一遍。" (I2)

Village doctors found it convenient to use the loudspeaker and thought that most villagers could hear the announcement (I1, 2, 6, 7). We found that the ability of the loudspeaker to make a clear announcement differed hugely per village; some loudspeakers did not work well, while others could reach the whole village. Also the effectiveness of the speaker in recruiting caregivers differed per village. In some villages, after making the announcement only one or two caregivers came to the village clinic, while in other villages many caregivers came. When parents did not hear the announcement, others told them or grandparents came.

Making phone calls. Village doctors (I3, I5) said that they tried to make phone calls to caregivers they knew when they had their phone numbers. Some village doctors called caregivers themselves and when they were not able or willing, we called caregivers.

Visiting caregivers' homes. One interviewed village doctor went from door to door to recruit caregivers (I6). Most village doctors did not visit caregivers' homes and it was mentioned that we did not ask for this (I2).

"VD: They did not ask me to take them to caregivers'… caregivers' homes, I…I did not do that.他们也没让说叫说让我领他们下去……去底下下户……入户啊，我我我……也没有再去。" (I2)

A number of village doctors who were not interviewed went from door to door. We felt that most village doctors did not go from door to door for the following reasons: the village was too big and it was impossible to visit each home as this took too much time, and village doctors were not familiar with all caregivers of eligible children and therefore they did not know the location of their homes.

Other recruitment methods. We went on the street to ask caregivers and asked caregivers to notify others. We went to places where caregivers could be found such as the local nursery, market, or temple. Sometimes the village doctor helped us with these activities. In one village (village 45) that did not have a village doctor, the bus driver helped us to find the chief of the village who looked through the name list. However, the chief said that there were no children younger than five on the list he could find. Therefore, we went on the street to ask women, but this was unsuccessful.

Village doctors' work. We found the following factors influencing recruitment: (i) duties, (ii) division of work, (iii) work load, (iv) gathering caregivers for vaccination, and (v) recruiting caregivers for previous studies.

Duties. Village doctor's work included treating patients and prescribing medicines (I3, 4). All village doctors used a mobile phone, and it was mentioned that it had to be switched on 24 hours a day (I3). Some village doctors were in charge of maternal, newborn and child health services. Once in a while they had to submit a name list of pregnant women or children to their supervisor in the township hospital and county hospital. Village doctors had to go from door to door for registering pregnancies, conducting postnatal pregnancy visits, and to gather caregivers for vaccination of their children. These duties were not perceived as being complicated (I4).

Apart from village doctors' health care duties, we found that village doctors were sometimes busy with other work. Village doctors received their income mainly from medicine prescriptions (regulated by a fixed list of medicines that village doctors could sell). They were usually not paid in other ways by the government and thus relied on patient fees, which were not always sufficient. Therefore, most village doctors did other work; for example one village doctor worked on the field (agriculture) and in a restaurant and another village doctor worked as a dentist.

Divison of work. Village doctors from three villages talked about the division of work (I3, I4, I5); one village had only one village doctor who did all the work (I5), while the other two villages (I3, 4) had three doctors in each village. In one village, the work of doctors was divided between the three doctors and the interviewed village doctor was in charge of maternal, newborn and child health care (I4). In the other village, the work was not divided and thus the village doctor was not in charge of this care (I3).

Work load. Village doctors did not have much work during the time of the study (I1, 2, 3, 8). They had an average of two to six patients per day and at most around twenty (I 1, 2, 3, 4, 5, 6, 7, 8). However, the number of patients per day differed during the year; there were fewer patients in summer and more in winter (I1, 2, 8). The main work took place in the morning and evening, and the afternoon was not perceived as busy (I3).

Gathering caregivers for vaccination. Village doctors helped with gathering caregivers for vaccination when caregivers missed the dates on which vaccination took place (I6, 7, 8). Village doctors informed caregivers about vaccination by making an announcement with the loudspeaker, sending text messages, making phone calls, and going from door to door. They made appointments with them to arrange a time for vaccination. However, similar to the experiences with the current study, village doctors could also not always find all the children on the name list. Caregivers took vaccination seriously, because they did not seem to mind about the immunization fees and usually came without a reminder (I8).

"VD： Sometimes, when it is time…it is time for vaccination, on a given day in a month, when it is time, they will come without reminder. 有时候到这个，到这个时候了，一个月的几号，到这个时候了以后，他自己就来了。" (I8)

Recruiting caregivers for previous studies. A number of doctors had no previous research experience (I1, 2, 4).

"DXZ: As for you, has it previously happened frequently that surveys like ours take place in your village? 那您像，像我们这样的项目在您这个村子做的多吗？

VD: No. 不多。

DXZ: No? 不多是吗？

VD: See, like yours…is my first time to take part in a survey. 像你们…这是第一次过来。

" (I7)

Other village doctors participated in a survey with a child health expert from Beijing in 2011 (who was part of our research team) (I1, 2, 4). They remembered that this expert was an “old” doctor who could explain knowledge of disease prevention and provide consultation. At that time, village doctors also notified caregivers and caregivers received a towel for participation. The difference was that children received a test (blood test for checking health) for participation in that survey, and village doctors were then able to recruit more caregivers than for the current study. Also, a village doctor took part in semi-structured interviews with caregivers, a survey that we conducted last year, and examinations for hypertension and diabetes (I4). We used our experiences with village doctors from previous studies to conduct the fieldwork for this study.

Village doctors' motivations. We found the following factors influencing recruitment: (i) understanding of the study, (ii) interference with work, (iii) money, (iv) work for villagers, (v) follow orders from township and county hospital doctors, and (vi) cooperate with research team.

Understanding of the study. Village doctors remembered that supervising health workers from the township and county hospital contacted them, but this did not seem sufficient to inform them well. We found that a number of village doctors (I1, 3, 6, 9) did not know what the aim of our research was.

"WW: Ok. Then do you know what we are doing? 哦，那您现在明白么？

VD: Ok, I did not know it. (laughing with sounds; seems to feel a little embarrassed) 哦，我也不是嘛明白。呵呵。" (I1)

Some village doctors said to only superficially knew that our study was about pneumonia and diarrhoea (I1, 8).

"VD: I do not remember. I thought the survey was to investigate something about child pneumonia, diarrhoea, I mean, I only know this. 我记不得。我觉着就是调查这研究孩子们这肺炎，腹泻，就是，我就光明白这一点。" (I1)

One village doctor (I4) thought the survey had no use, because not all the questions we asked were relevant.

"VD: How to say… for example, when caregivers came, you just asked questions about whether their child had cough, was ill, had pneumonia or had diarrhoea. And, and that…you only asked those questions, nothing else. Nevertheless, you asked about the family situation, incomes or expenditure and so on. I think this is not much use. 怎么说呢，你看，来了吧之后就是问一些孩子有没有咳嗽呀，生病呀，肺炎呀，腹泻

呀。另外，另外就是……问问而已，别的事情也没有，另外在问问人家家庭情况，收入啦或者花销之类的，我感觉这个没什么多大用。" (I4)

Only two village doctors had a good understanding of the research (I5, 8).

"VD：But, in my mind, it does not matter how much, we are in the same county, I will cooperate with you. Sometimes the work cannot be done by only one doctor. For your science research, many people will take part in it. 但是呢要我说，多跟少都无所谓，咱是一个县的，协助工作。有时候呢不是你一个医生能完成的。你搞这科研吧。不定参与多少人呢。。

DXZ：Yes, exactly. 恩，是是是。

VD：We cannot deal with that (to reach the aim of the research). And the result comes, when the result comes, when it is time to use the result, we can share the benefit. 那不是咱能解决的，以后他得到的结果，有成果以后，他利用成果的时候

咱就受益了。"(I8)

Interference with work. At the time of the current study, many village doctors said to not be busy and that the study did not interfere with their normal work. However, when it was the busy season, our study would delay village doctors' work and then they were not able to help us.

Money. Village doctors did not give a clear answer about what they thought about the money we gave them and said that they did not mind about payments (I1, 2, 3, 4, 5, 7, 8).

"WW: I mean actually we… want to ask, was the compensation we provided you each time, suitable? 就是我们。。。其实还想问一下，就是说我们每次给您的劳务是不是合适？

VD: Ah! It does not matter! 哎！那有嘛合适不合适！" (I1)

Two village doctors (I7, 8) said that because they understood the aim of the survey, they did not mind about the payments. If it could improve children's health conditions, they were willing to cooperate.

"DXZ: Yes. And I want to ask you that how do you feel, I mean…the compensation we gave you. How do you feel about…对是是是。那我还想问一下，就是您觉得。。。就是我们给您的劳务费，您觉得怎么样？就是您觉得。。。

VD: I do not mind about that. 那倒无所谓。

DXZ: You do not mind about that? 那无所谓？

VD: No, I do not. It is ok whether you give it to me or not. It does not matter, because we undertook the work together and for the benefit of the children. It does not matter, that is it. 恩，那无所谓。你给也行，不给也行，那个倒无所谓。因为咱这工作都是合作的，都是为了儿童好。那个倒是小事，那个。" (I7)

Village doctors in clinics with more than one village doctor had to share the money with the other doctors, which considerably decreased the amount of payment. However, not more caregivers could be found if we gave them more money (I1).

"VD: If you give (me more money)…then it is the same, I cannot find them. 要是给。。。那样也是找不着。" (I1)

A village doctor (I2) thought that the payment was ok, while some (I4, 5, 6, 8) thought it was too little considering their regular income, especially when they had a large village. One village doctor complained during the first and second visit about payment (I6). Village doctors usually received more payment for other tasks that required similar efforts and time, and considered the amount of ¥ 50 to be small.

"VD： The difference is huge. 这没法比。

DXZ： Ok. The difference is huge. 哦 没法比。

VD： Sometimes, one treats patients for half a day, there can be more than ten patients. That is our regular income, not including the cooperation medical (insurance) scheme, even if it was only my regular income, it will be much more than that.

有时候，你看半天病吧，你看十多来个病人，咱正常的收入啊，咱不说合作医疗什么的，正常收入，要比那个多的多。” (I8)

Village doctors also perceived the payment as little, because when they missed patients during time in which they worked for the study, patients would go somewhere else for treatment (I4, 5, 6, 8). Then village doctors would not only miss payments on that day, but also future payments as patients often had to be treated for several days and paid on each day.

We found that some of the village doctors seemed to mind about the study compensation. Only one village doctor in a large village was able to recruit about 70 participants during the first recruitment visit and received more than ¥ 50 (village 42). We only had a note of ¥ 100 and told the village doctor that we paid ¥ 70 and asked for change. However, the village doctor said to deserve ¥ 100 for the work and did not return the ¥ 30 change. For the second visit, increasing the incentive to ¥ 10 for every four participants village doctors recruited did not result in recruitment of significantly more caregivers. Only the village doctor who recruited about 70 caregivers during the first visit (village 42) was able to recruit a considerable number of participants and again wanted ¥ 100 for the work.

Work for villagers. Most village doctors gave the impression that they cared about villagers and wanted to help them. A village doctor said to have chosen this job taking in consideration that it could help villagers (I10). Some village doctors said that they liked to help with the study, because it did good things for children's health (I4, 7, 8, 10). They were more positive about the study when they thought that it was good for children (I4).

"VD: …It's not….it's not meaningless, I mean that...as long as it does something for villagers or children, no matter what it is, I can work for it. I can…such as help you with participation or coordination or whatever. Some things, if it is not meaningful, I will…take it, anyway, …slightly….less seriously.

不是。。。不是说没意思，我的意思是什么。。。只要对乡亲们或者孩子好，不管怎么样，我都可以这个。我都可以说。。。帮忙说参加或者组织或者干嘛，都可以。有的事情，如果说没多大意思或者什么，我就。。。反正还是稍微。。。怠慢一点" (I4)

Follow orders from township and county hospital doctors. Village doctors said that they would finish the tasks even when there were difficulties, because they were being given orders from supervising staff from the township and county hospital. It did not matter what the tasks were, whether they had payments or time for meals, they would finish because they had to (I1, 5, 6).

Cooperate with the research team. Some village doctors said that they wanted to work for us to help us with coordination and to make our work easier (I5, 7, 8).

Caregivers' characteristics. We found the following factors influencing recruitment: (i) education and (ii) ability to text message.

Education. Village doctors said that caregivers' education levels were relatively low (I4, 8). Village doctors thought that it was not useful to ask caregivers with a lower education all questions, because there were many questions they could not answer.

"VD： The key thing was that they did not know what you were asking, like this, it was useless to ask them. 你问题好多事他不知道说嘛了，你像这样的就白说跟他。

DXZ： Useless. Ok. 白说，哦。

VD： And, then, their education level is comparatively not so good, it is a little low. 恩，其次呢，比较，文化水平也不占，也比较落后一点。" (I8)

Our experiences were that most caregivers could understand the majority of questions, were likely to tell us the truth and keen to talk to us about their child. On rare occasions, we could judge from the way a caregiver acted in the interview that the caregiver truthfully answered the questions. Sometimes grandparents had difficulty with understanding our survey questions, because their education was generally lower than parents' education.

Ability to text message. Parents could usually text message, but grandparents were often not able to text message as many of them had not learned how to use the pinyin system, which was required to type a text message. In China, it is very common that grandparents take care of their grandchild(ren). Therefore, when parents could not come to the village clinic, grandparents came. Grandparents liked to receive the towel for participation. Grandparents sometimes said "I am taking care of the child, why don't you ask me?". We tested grandparents' text message skills to ensure that we only included grandparents who could text message. Sometimes grandparents tried asking others to help them with replying so that they could participate and receive the towel. When we doubted their text message ability, we would test them again and carefully watched them typing the text message.

Caregivers' motivations. We found the following factors influencing recruitment: (i) understanding of the study, (ii) interference with work, (iii) trust, (iv) sensitive questions, (v) reward (towel), and (vi) health information.

Understanding of the study. Some village doctors (I1, I3, I5, I9) recalled what they said to caregivers about the survey, but this often seemed not very precise.

"VD: Children under five, eh…let their parents come to the clinic with their child the next day. Someone from Capital Institute of Paediatrics is coming to check health service. 5岁以下嗯。。。父母带着孩子第二天来卫生室，有北京儿童研究所来调查来了工作"。" (I3)

However, some of them gave a more informative explanation.

"VD: When you first came, you said…at that time.. this is a good thing. I told them if you come, you can get a towel as a reward. 头一次过来那。。你说你说那时候。。。这事就是好事，我跟他们说来了到时候给你块毛巾，有奖励。

WW: Yes. Yes. 恩恩。

VD: See. I said this is not other things, it is a …eh…for the nation, it is not misinforming. Right? 是吧。我说这不是别的，这是国家的一个那什么。。。这不是别的，不是骗咱，是不是？" (I5)

As a result, caregivers did not always understand the aim of the study and thought that it was not useful to come to the village clinic to participate when their child was usually not ill. Caregivers' then did not consider participation in the study a priority.

"DXZ: Did caregivers not come for their child's health? 那不是他们家孩子的健康问题吗？

VD: It did not work to tell them it is about health. 健康问题也不行。

DXZ: It did not work? 也不行？

VD: Some children, they do not get ill, right? 有的孩子吧，人家不好得病，对吧？

DXZ: Ok. They do not get ill, so they do not come, they did not come because their children did not get ill, right? 哦，人家不好得病，所以就不来，没生病他就不来了，是么。

VD: Yes. 对。" (I6)

We carefully told the aim of our study to caregivers who came to the village clinic, but village doctors thought that our explanation to caregivers was not sufficient for them to understand the research.

"VD: Even when you came that day, you told them, but they did not understand either. 就是那天你们来了，你给他们讲，人还是不明白哪都。" (I1)

However, our experiences were that while some caregivers could not understand the study well, most gave the impression to have a good understanding.

Interference with work. A village doctor said that caregivers sometimes did not want to participate, because they had much work to do. Some caregivers had to work hard (long days and no rest days) in local factories, but then earned a relatively high salary (¥ 3000 per month) (I8).

"VD：… you see, they earn ￥100 (per day), and you asked them to waste some time to talk about children's health, they had no time. They did not want to come.。。。人家一百多块钱挣着，你说叫他耽误工夫把孩子情况说说，没工夫，他不愿意来，没工夫来。" (I8)

Trust. We carefully explained caregivers that we were not there to misinform or delude them, but found it not so easy to convince caregivers to trust us. Village doctors felt that caregivers did not have a good understanding (I1, 3, 8, 9) and still were afraid of being misinformed or deluded (I3, 9). Some caregivers had experienced being cheated in the past and therefore were afraid that it could happen again. Other caregivers had heard of cases where people had been cheated on television and were therefore apprehensive (I9).

Sensitive questions. Village doctors thought that questions about income and expenses were sensitive to caregivers (I4).

"VD: You see, briefly… you see…you are…you said you study children's diseases, such as cough and diarrhoea. That is part of your study. I did not mean simple questions and so on, that you asked. But how about the questions about parents' income and expenditure that you asked, what are the aims of asking those questions? It has nothing to do with children. Why did you ask that? 你看，你简单吧，你看啊。。。你是什么。。。你说调研儿童的疾病或者什么的之类的，咳嗽啦，腹泻啦，这是一方面，你说了，不是说简单或者别的怎么说，你都是问了，但是后面家长的收入啦，花销啦这个，光问那有什么意思啊，根本跟儿童都是没有任何关系的，你问人家那有什么意思。"(I4)

We also found that caregivers asked us about the purpose of some questions and then we would explain the reason for asking those questions.

Reward (towel). Village doctors thought that the reward (towel) worked well to recruit caregivers (I1, 5, 6, 7). Village doctors said that only telling caregivers about the importance of participating for child health was not sufficient to convince many caregivers to participate (I6, 7, 8). Most village doctors thought that caregivers would only come to the village clinic if we told them they would receive a reward (I6).

"DXZ: Ok. Then what is typically said to them so that they came? 哦那您一般都怎么跟他们说他们才能来啊？

VD: Sometimes, you see, (you can tell them) we did not ask you to come for nothing, there is a reward for you. 有的时候，你看，你去了到时候不叫你白去，是吧，给你点东西。

DXZ: Ok. Tell them there is a reward. 啊，说给点物件。

VD: Villagers think in this way. 底下老百姓都是这样的心理。"(I6)

However, also it was also mentioned that some caregivers did not participate for the reward and that they would participate even without the gift (I7).

“VD: They are definitely not only here for the towel, if there is no towel, some of them will still come. 他不一定就说为毛巾来，你就说不送，他该来的照样还是来。”(I7)

Our experiences were also that while a small number of caregivers only participated for the towel, most did not, though they appreciated the reward.

Health information. Based on our experiences, participants seemed more interested when they could obtain health information. A village doctor said that it better to tell caregivers that “child health experts” came to their village and that they could learn something from coming to the village clinic (I4). However, we could not give participants much health information due to the lack of time and expertise. The infant feeding calendar that we gave may have encouraged some caregivers to come to the village clinic, but this did not increase caregivers’ willingness much, because the calendar was from the previous year (2012).

Recommendations for ways to recruit more caregivers

Table S2 presents village doctors' and our recommendations for ways to recruit more caregivers. In the following sections, we describe these recommendations in detail.

Table S2. Village doctors' and our recommendations for ways to recruit more caregivers

Village doctors' recommendations	Our recommendations
Give name list and phone numbers in advance	Good strategy
Include all caregivers	Not feasible
Visit villages earlier on the day	Good strategy, in addition villages could be visited in the evening, but working hours of village doctors and interviewers need to be taken in consideration
Continue using the villages' loudspeakers	Good strategy
Continue making phone calls and send text messages	Good strategy
Give village doctors more time for visiting caregivers' houses	Feasible when village doctors know where caregivers live
Give caregivers money	Not desirable and not feasible
Give caregivers' children a free health test	Only appropriate when this is required for the study
Bring a doctor for free consultation	Possible strategy for increasing trust, but explore option of sending health information text messages
-	Develop and test new information materials for village doctors and caregivers
-	Omit sensitive questions from survey
-	Tailor recruitment strategy to the specific context of villages

Giving name list and phone numbers in advance. A village doctors said that giving the name list beforehand was better, because this allowed them time to find out where the children lived (I2). Some mentioned that it would be better to inform caregiver in advance, such as on the previous evening or at least one hour before the survey, because this gave caregivers a chance to ask for leave from work (I6, 7, 8).

"VD： When you called them to come (announcement), they left. You should tell them in advance. There are rules in their company; it is not convenient to ask for leave. 你一喊他的时候他就走了。你提前喊，人家上班都有纪律，都有要求，人家请假也不好请。" (I4)

We think that this could be a good strategy to facilitate recruitment. Caregivers could be sent text messages in advance and be called at a time nearer to the recruitment.

Include all caregivers. Village doctors said that if we would include all caregivers, then they would be able to recruit more caregivers (I1, 3, 8).

From our perspective, the selection criteria mainly restricted grandparents from participating in the study, because they were often not able to text message. However, for interventions that involve text messaging, this selection criterion is essential.

Visit villages earlier on the day. Village doctors thought that it would be better recruit caregivers earlier on the day, because then more caregivers had time (I2, 7). This would have to be early in the morning before caregivers went to work and children woke up; around 7.30/8 am in summer.

We think that this could be a good strategy, but that recruiting caregivers in the evening after 7 pm when caregivers come home may be useful too. However, we have to take into consideration that village doctors also said to be busy with work during in the morning and evening, and interviewers would have different working hours.

Continue using the villages' loudspeaker. Village doctors were used to making announcements by loudspeakers and found this the easiest way to recruit caregivers (I2, 6, 7). They liked using the loudspeaker in the morning and evening, because caregivers were then at home (I6, 7, 8). It would be best to use the father's name (I2). Fathers usually lived longer in the village than mothers and therefore more villagers knew the father's name. When the mother's name was used, villagers and village doctors often did not know who the mother was.

"VD: You see. You read the fathers' names with the loudspeaker and others living there... I mean if others hear that, not the father, they will tell him, eh…like the speaker called you to the clinic or somewhere. They will know. If you call the mothers' name, they do not know. 是吧，你广播了爸爸，大部分那住的……就是，别人……人听到了，你听不到，也能转告你。恩。。。喇叭喊你咧，叫你去干什么什么的，去卫生室，或者去哪块儿咧人家都知道，你有时候叫那个女的的名，人家都知不道。" (I2)

We think continue using the loudspeaker in the proposed way is a good strategy.

Give village doctors more time for visiting caregiver's houses. Village doctors were willing to notify caregivers by going to their homes (I1, 3, 6, 7), but this approach was not ideal, because, it was much work, they did not know all the houses and caregivers sometimes lived far away (I1, 3, 6). Therefore, village doctors only wanted to go from door to door when the loudspeaker did not work well (I2), they were not busy (I3) and knew where caregivers lived (I6). More time was needed to be able to do this (I6). However, caregivers could still not come when they were not home (I1).

"VD: It is that they lived far from each other. Sometimes when you call them (by going to their home), it is a waste of time. For instance, you go to someone's house and nobody is here. They are usually at home in the evening.

他这个就是有的时候住的太分散，有时候叫或者是喊的话，就浪费时间。你比如说去了人家，家里没人，一般晚上都在家。” (I4)

From our perspective, going from door to door would be a very costly and time-consuming approach, but could be used when village doctors know where caregivers live.

Continue making phone calls and send text messages. It was mentioned that it was a good approach to call caregivers or send text messages to them and that this should be used in future studies (I2).

We think continue making phone calls and send text messages is a good strategy.

Give caregivers money. If the amount of money caregivers received for participation would be more than their salaries, then caregivers would come (I8).

"VD：To say it honestly, it is, if they did not work outside, if you can give a similar profit to their work, they can thus stay here and help you. 说直接点，说白了就是，他如果不去打工了，他能够得到跟他打工相当的好处，他能不出去，这个他就可以在这我给你看着。” (I8)

We think that this would not be a suitable strategy, because it may result in recruiting caregivers who only participate for the reward and we would not be able to afford paying all caregivers a considerable amount of money.

Give caregivers' children a free health test. It would be better if there was a free test for children, such as an anaemia test, or micro-nutrition test. This was perceived as good and would be an incentive for caregivers to come to the village clinic (I4).

"VD: You…you are, after all, not professionals (medicine). You are not a doctor. You are people that research. You can ask their symptoms and so on, it is ok. But I feel it is not a professional thing, not like with a specialist who treats an ill child, certain symptoms means certain illnesses, or certain types of checkups which can improve health. These activities I think are acceptable.

你们。。你们毕竟不是专业的，不是医生，调研的就只是调研的，问问他们症状啊什么之类的，还行，但是说，毕竟不是专业的东西我感觉，不是说专家来，孩子要生病了，什么样的症状就是什么样的病，或者就检查什么之类的，可以促进一下这我感觉还行。” (I4)

We think that giving a free health test for children should only be used when this is relevant to the study.

Bring a doctor for free consultation. It was mentioned that we were not clinical doctors, we did not know about diseases and we could not answer caregiver's questions about diseases well (I4). It was found better to bring an experienced doctor, give health education and answer caregivers' questions.

"VD: To say it simply, I mean, you must understand that… for example, you can bring a book about child disease prevention. When you come, regardless of whether they have questions or not, you can tell them, what they need to pay attention to with the child, what symptoms may imply what kind of disease, I think it is better to tell them like this kind of information. 简单的意思就是什么，你必须得搞清楚或者比方说什么……你可以拿一本儿童疾病什么防疫，还是什么，来了之后吧，人家比如说有了或者没有了，你可以简单的跟人家说，以后孩子要注意什么，什么又什么样的症状就是什么样的病，这样给人家一说，我感觉好一点。" (I4)

We felt that there was a great need for health information. Bringing a clinical team member could be a good strategy to increase trust. However, using text messaging to disseminate health information may be a more cost-effective solution.

Additional recommendations from researchers. Moreover, to address factors that negatively influenced recruitment, we suggest to develop and test new information materials for village doctors and caregivers, omit sensitive questions from survey and tailor recruitment strategies to the specific context of villages.

Table S3. Characteristics of interviewed village doctors and villages, and experiences with recruitment in group 1

						Recruitment visit 1							Recruitment visit 2			
Village number	Interview village doctor	Gender and age interviewed village doctor	Category of number of villagers*	Category of number of children <5†	Total number recruited	Month. Day	Loud speaker used?	Phone numbers provided by township?	Phone numbers provided by village doctor?	Other method used?	Cooperative village doctor?	Comments	Month. Day	Loudspeaker used?	Other method used?	Comments
1	-	-	3	2	15	3.17	No, not available	Yes, 53	Yes	No	Yes	Village doctor made all phone calls.	3.25	No	Call	Not very effective recruitment
3	-	-	4	2	13	3.16	No, not available	Yes, 33	No	No	Yes	Some children were not from this village, possibly mistakes in the name list. Village doctor took us to caregivers' houses.	3.24	No	Call 32	-
4	I6	M, 49	3	4	21	3.16	No, not available	No	No	No	No	Village doctor complained about payment. Some children were not from the village, may be mistakes in the name list.	3.22	No	No	Village doctor again complained about payment.
10	-	-	3	4	18	3.16	No, not available	No	No	A notice near a temple, we asked caregivers to ask others	Yes	Most of the mothers worked as guide around the temple, village doctor wrote down the information on a notice board and also asked them to tell others. Right time for recruitment (4 pm).	3.25	No	No	Village doctor was busy with patients and not willing to help.

12	-	-	3	3	15	3.16	No, not available	No	No	Village doctor went to homes	Yes	Difficult to recruit, because large village, but no loudspeaker or phone numbers available.	3.26	No	Village doctor took us to homes	-
13	I2	F, 40	3	3	43	3.16	yes	No	No	No	Yes	Village doctor asked many eligible parents to come.	3.22	No	Going to houses and asked people on the street	Village doctor helped to visit houses and also asked caregivers on the street.
16	-	-	2	1	15	3.17	Yes	Yes, 28	Yes	No	Yes	Village doctor helped checking the vaccination card of caregivers (used for identification).	NA	NA	NA	No second visit, all efforts tried in first visit
17	-	-	3	2	27	3.17	No, not available	Yes, 78	No	Village doctors asked on the street and went to houses	Yes	Village doctor had experience with gathering caregivers. We could not finish calling all caregivers.	3.24	No	Call 36	Completed calling all phone numbers
20	I7	M, 63	4	3	47	3.17	Yes	Yes, 101	No	No	Yes	Good quality loudspeakers, village doctor made announcement several times. Did not have time to call all phone numbers.	3.22	Yes, twice	Call +asked caregivers to ask others	Village doctor said to not mind about the money and was willing to do everything to help
27	-	-	2	2	4	3.17	Yes	No	No	No	No	Walked to village because of road works. Based on previous experience we expected recruitment to be	3.25	NA	NA	Not able to visit village, road completely blocked.

												difficult. Scheduled recruitment in late afternoon, but not successful. Loudspeaker not of good quality. Village doctor not willing to call caregivers and was busy (had to pick up child). Found mother from this village in another village.				
32	-	-	3	4	41	3.16	Yes	No	Yes	Asked caregivers to as others	Yes	Village doctor made calls.	3.24	Yes	No	Village doctor was busy and not able to go to houses. Was not as cooperative as during first visit.
36	-	-	2	2	33	3.17	Yes	No	No	No	Yes	Village doctor made announcement before we came.	3.25	No	Went to the homes	-
40	I4	M, 29	4	5	31	3.16	Yes	No	No	No	Ok	Initially, we did not give the name list. Village doctor used own money to make the announcement with private loudspeaker. However, village doctor did not want to go to homes. Village doctor participated in previous research, but thought this was not useful or helpful for mothers.	3.23	Yes	We asked people on the street	We gave the name list. Right time of visit (10 am) and better recruitment.

43	I8	M, 57	4	3	37	3.21	Yes	No	No	Went to market to ask people	Yes	It was market day and the market was just outside the village clinic. Village doctor wanted more payment.	3.24	Yes	No	-
45	NA	-	1	1	0	3.21	No, broken	No	NA	We asked people on the street	NA	Village doctor passed away. Village chief went through name list, but could not find caregivers.	NA	NA	NA	NA
46	I9	F, 40	2	2	11	3.16	Yes	No	No	We asked people on the street	Yes	Initially, we did not give the name list. Loudspeaker was not of good quality. Villagers neither trusted us nor the village doctor, despite explanation. Village doctor said most of the villages were doing business on their own and therefore recruitment was hard. Villagers feared being deluded, because they saw this on television (they were not misled). Village doctors asked for more payment, even after explanation. There was no other study before. Village 39 is next to this village, but recruitment was much better there.	3.21	Yes	We asked people on the street	One caregiver could not understand the study. One caregiver said that the grandparents took care of the children and we should ask them. Caregiver feared being deluded.

*Number of villagers:1=<500, 2=500-1000, 3=1000-2000, 4=>2000

†Number of children under five: 1=<50, 2=50-100, 3=100-150, 4=150-200, 5=>200

Table S4. Characteristics of interviewed village doctors and villages, and experiences with recruitment in group 2

					Recruitment							
Village number	Interview village doctor	Gender and age interviewed village doctor	Category number of villagers*	Category number of children <5†	Total number recruited	Month. Day	Loud speaker used?	Phone numbers provided by township?	Phone numbers provided by village doctor?	Other method used?	Cooperative village doctor?	Comments
2	-	-	2	1	17	3.18	No, not available	Yes, 23	No	No	No	Village doctor was also a dentist and too busy during the time we were there.
5	-	-	3	2	7	3.19	No, not available	Yes, 53	No	Village doctor made phone calls on day before recruitment	Yes	Village doctor said to not know some of the children on the name list and maybe some of them were not from this village.
6	-	-	2	1	0	NA	NA	NA	NA	NA	NA	Township doctor said that the village did not have a village doctor.
7	-	-	3	2	11	3.21	Yes	Yes, 45	No	No	Yes	Village doctor helped calling and asked people to inform others.
8	-	-	2	1	24	3.21	Not sure; late arrival	Yes, 34	No	No	Yes	Communication problem resulted in late arrival. There were many caregivers (mainly grandparents) waiting for us and took much time to comfort them. We did not have time to call phone numbers.
9	-	-	2	2	23	3.18	No, not available	No	No	No	Yes	Difficult to recruit caregivers, because there was no loudspeaker
11	-	-	3	5	16	3.18	No, not available	No	Yes	Asked caregivers to ask others before we arrived		We asked caregivers to ask others. Village doctor made phone calls. Recruitment was better than for a previous study.
14	-	-	3	2	0	3.18	Yes	No	No	No	No	Village doctor was too busy with patients and prescribing medicines and not willing to help. We made the announcement; only two mothers came to the clinic, but could not text message.
15	-	-	3	3	7	3.20	Yes	No	No	No	Yes	Village doctor knew almost all parents and knew if they were going to work or were at home.
18	-	-	2	1	12	3.20	Yes	Yes, 32	No	No	Yes	We called 27 phone numbers, but could not call the last 5.

19	-	-	2	1	14	3.20	Yes	Yes, 17	No	No	Yes	We called all phone numbers.
21	-	-	3	3	24	3.20	Yes	No	No	No	Yes	We made the announcement several times.
22	-	-	2	2	10	3.20	Yes	No	No	Asked caregivers to ask others	Yes	Village clinic was close to a nursery where we asked caregivers.
23	I10	F, 41	3	3	15	3.21	Yes	No	No	Asked caregivers to ask others	Yes	Village doctor helped us to explain the study to the caregivers and asked them to tell more people to come.
24	-	-	3	2	24	3.19	Yes	No	No	No	Yes	-
25	I3	M, 38	3	3	4	3.19	Yes	No	No	Went to nursery and asked on the street	No	Village doctor was drunk when county hospital doctor made visit. Teacher in nursery said village doctor "was not good". Village doctor gave the impression to lie during interview.
26	-	-	2	2	12	3.19	No, not available	No	No	Asked caregivers to ask others	Yes	-
28	-	-	3	3	16	3.20	Yes	No	Yes	No	Ok	We made the announcement and made phone calls.
29	I5	M, 59	4	5	36	3.20	Yes	No	No	Village doctor went to homes	Yes	Village doctor wanted to earn more money and went to homes, but did not find caregivers.
30	-	-	4	5	0	NA	NA	NA	No	NA	NA	Village doctor was not available for a personal reason.
31	-	-	2	4	34	3.20	Yes	No	No	No	Yes	Village doctor asked many eligible parents to come. Village doctor said caregivers lived far away from the clinic and was busy and not willing to take us there.
33	-	-	3	4	38	3.20	Yes	No	No	No	Yes	Village doctor asked many eligible parents.
34	-	-	3	4	31	3.19	Yes	No	No	No	Yes	Village doctor asked many caregivers. When we got there, many parents were waiting for us.
35	-	-	3	3	43	3.19	Yes	No	No	No	Yes	Village doctor was nice and asked lots of eligible parents to come.
37	-	-	3	4	47	3.19	Yes	No	No	No	Yes	Village doctor asked many eligible parents to come.
38	-	-	1	1	15	3.19	Yes	No	No	No	Yes	Village doctor knew villagers well.
39	-	-	3	3	19	3.18	Yes	No	No	No	Yes	-
41	-	-	3	3	38	3.18	Yes	No	No	No	No	Village doctor was old man and not willing to visit houses; reluctant to help.
42	-	-	4	5	89	3.17	Yes	No	No	Asked caregivers to ask others	Yes	Village doctor's partner helped to make the announcement and many eligible mothers came. Village doctor said caregivers received health information calendar last year.
44	I1	M, 38	2	2	17	3.18	Yes	No	No	Asked caregivers to ask others before we arrived	Yes	Several persistent grandmothers, despite careful testing there was potential risk that they could not text message.

*Number of villagers:1=<500, 2=500-1000, 3=1000-2000, 4=>2000

†Number of children under five: 1=<50, 2=50-100, 3=100-150, 4=150-200, 5=>200
